# Reconstructing Mammalian Phylogenies: A Detailed Comparison of the Cytochrome *b* and Cytochrome Oxidase Subunit I Mitochondrial Genes

**DOI:** 10.1371/journal.pone.0014156

**Published:** 2010-11-30

**Authors:** Shanan S. Tobe, Andrew C. Kitchener, Adrian M. T. Linacre

**Affiliations:** 1 Centre for Forensic Science, WestCHEM, Department of Pure and Applied Chemistry, University of Strathclyde, Glasgow, United Kingdom; 2 Department of Natural Sciences, National Museums Scotland, Edinburgh, United Kingdom; 3 Institute of Geography, School of Geosciences, University of Edinburgh, Edinburgh, United Kingdom; 4 School of Biological Sciences, Flinders University, Adelaide, Australia; American Museum of Natural History, United States of America

## Abstract

The phylogeny and taxonomy of mammalian species were originally based upon shared or derived morphological characteristics. However, genetic analyses have more recently played an increasingly important role in confirming existing or establishing often radically different mammalian groupings and phylogenies. The two most commonly used genetic loci in species identification are the cytochrome oxidase I gene (COI) and the cytochrome *b* gene (cyt *b*). For the first time this study provides a detailed comparison of the effectiveness of these two loci in reconstructing the phylogeny of mammals at different levels of the taxonomic hierarchy in order to provide a basis for standardizing methodologies in the future. Interspecific and intraspecific variation is assessed and for the first time, to our knowledge, statistical confidence is applied to sequence comparisons. Comparison of the DNA sequences of 217 mammalian species reveals that cyt *b* more accurately reconstructs their phylogeny and known relationships between species based on other molecular and morphological analyses at Super Order, Order, Family and generic levels. Cyt *b* correctly assigned 95.85% of mammal species to Super Order, 94.31% to Order and 98.16% to Family compared to 78.34%, 93.36% and 96.93% respectively for COI. Cyt *b* also gives better resolution when separating species based on sequence data. Using a Kimura 2-parameter *p*-distance (x100) threshold of 1.5–2.5, cyt *b* gives a better resolution for separating species with a lower false positive rate and higher positive predictive value than those of COI.

## Introduction

Species classification depends on our understanding of morphology, behavior, ecology and genetics of organisms. Taxonomy and systematics are dynamic disciplines, changing frequently owing to new evidence and changing consensuses on species definitions. Species boundaries and higher taxonomic categories within the Mammalia are historically based on morphological characteristics (e.g. [1]–[6]). More recently genetic comparisons have led to greater understanding of lineages of related species, especially at higher taxonomic levels, where derived morphological characteristics can be difficult to determine owing to ancient divergences, thus leading to often radically different phylogenies and species groupings [1], [7]–[13]. In recent years several molecular and combined molecular and morphological studies have confirmed the presence of four main Super Orders, the Afrotheria, Xenarthra, Laurasiatheria and Euarchontoglires, which have radically superseded previous taxonomic groupings [1], [9], [10].

The genetic loci of choice for many taxonomic and phylogenetic studies are primarily found on the mitochondrial genome [14]. Within mitochondrial DNA (mtDNA) some gene sequences are thought to exhibit little intraspecific variability, but show sufficient interspecific variation to allow for estimation of degrees of relatedness and divergence times via calibrated molecular clocks. Studies have used many different loci on the mitochondrial genome such as 12S rRNA (e.g. [15], [16]), 16S rRNA (e.g. [17], [18]), COII (e.g. [19]–[21]) and others. However, the main locus used in species discrimination until recently was cytochrome *b* (cyt *b*) [22], [23], which occurs between bases 14,747 and 15,887 in human mtDNA [24], [25]. More recently use of cytochrome oxidase subunit I (COI) has increased owing primarily to its adoption by the Barcode for Life Consortium [26], [27]. COI is found between bases 5,904 and 7,445 in human mtDNA [24], [25]. No previous study has quantified intraspecific variation in these two loci and made direct comparisons of their effectiveness in reconstructing mammalian phylogeny, although a few previous studies have investigated a limited number of species or gene fragments for particular Orders (e.g. [28]–[31]).

Being able to diagnose species and determine interspecific relationships are of primary importance in biology, ecology, evolution, systematics, wildlife management, conservation and forensic science. Typically phylogenetic studies depend on sequencing one or, more usually, part of one of COI or cyt *b*, followed by comparison with DNA sequences held on databases (e.g. EMBL or GenBank) [32]. These comparisons assume that registered sequence data are: i) correct and the sequence is not from another species or contains errors; ii) diagnostic for each representative of the species rather than being a rare example of subspecies or individual variation; iii) and that all individuals of a species have identical, or very similar, DNA sequences. It would be expected that two members of the same species have nearly 100% identity at either cyt *b* or COI. If the identity match is less than 100% then either there is some intraspecific variation, or the compared sequence comes from an unknown, but closely related, species. Although the degrees of intraspecific variation and divergence between closely related species have been investigated by some authors (e.g. 7.93% between and 0.43% within bird species for COI [33]; 5.7% between and 1.5% within *Stenella* species for cyt *b* [22]), these are generally poorly studied.

In addition many authors construct phylogenetic trees from sequence data on the assumption that any tree based on genetic data is the ‘true’ evolutionary history of those organisms (e.g. [33]–[35]). When anomalies arise, these are often interpreted as cryptic species [33]. However, cryptic species may be designated based on levels of expected intraspecific variation observed between as few as two individuals [36]. Currently the use of multiple genetic loci to infer phylogeny is routine (e.g. [37], [38]). However, if a single gene could be used this would be beneficial because it would standardize the loci used, and reduce cost, time and complexity of comparisons.

This study aims to compare entire sequences of COI and cyt *b* from the same individuals to assess patterns of variation within and between different mammalian species and to see how these relate to their evolutionary histories. Specifically we wish to (i) identify if either gene sequence can be used to reconstruct mammalian evolutionary history and if so which one does this more accurately; (ii) determine levels of variation within each gene between different mammalian species; (iii) determine levels of intraspecific variation within COI and cyt *b* and; (iv) identify which gene provides the greatest power in distinguishing between closely related species. For the first time this study will provide an unbiased analysis of both genes using the same criteria for each and will make recommendations based on their use in phylogenetic reconstruction and species discrimination in mammals.

## Materials and Methods

### 2.1 Sequence Data and Alignment

All sequence data were obtained from GenBank on the NCBI website (http://www.ncbi.nlm.nih.gov/). For the interspecific comparisons of cyt *b* and COI genes, whole mitochondrial genome sequences from 236 mammals (comprising 29 Orders, 89 Families, 174 genera and 217 species) were obtained; a full list can be found in Table S1 with references in Text S1. The cyt *b* and COI genes were isolated from the complete sequences for alignment. It was assumed that the sequences were correct and that species designations were accurate, although it is possible that errors may have occurred.

Complete mitochondrial genome sequences were obtained for 945 humans, *Homo sapiens*, 130 domestic cattle, *Bos taurus*, and 35 domestic dogs, *Canis familiaris*, to assess intraspecific variation (Table S2).

Sequences were aligned using ClustalW in the Molecular Evolutionary Genetics Analysis (MEGA) software package version 4.0 [39]–[41] on a desktop PC. Pairwise and multiple alignment parameters were gap opening penalty 15; gap extension penalty 6.66; delay divergent sequences 30%; DNA transition weight 0.5; and no use of a negative matrix.

### 2.2 Phylogenetic Trees and Analysis

Phylogenetic trees were constructed for cyt *b* and COI sequence alignments using the Maximum Parsimony, Neighbor-Joining, Minimum Evolution and Maximum Likelihood methods. The Maximum Parsimony, Neighbor-Joining and Minimum Evolution methods were calculated in MEGA 4.0 [39], [40], [42]. Maximum Likelihood trees were calculated using RAxML 7.2.3 [43] and MrBayes 3.1.2 [44]–[48]. The trees were then exported as Newick files and edited online, to assign color ranges using the Interactive Tree Of Life (iTOL) [49]. Each taxonomic Order was assigned a different color. Analysis of the phylogenetic trees showed that overall the Maximum Likelihood phylogenetic trees compiled using MrBayes showed the highest congruence with conventional taxonomic species groupings (as described below in 2.2.1), so these were used as the phylogenetic trees for detailed comparison.

The Maximum Parsimony trees were calculated using the complete deletion option, all codon positions and a CNI level of 3 with an initial tree by random addition of sequences (10 replicates). The Neighbor-Joining trees were calculated using complete deletion, all codon positions, a Kimura 2-parameter model and a CNI level of 1. The Minimum Evolution trees were calculated using complete deletion, all codon positions and a Kimura 2-parameter model, including transitions and transversions. All trees were calculated using 1,000 bootstrap repetitions and a random seed.

Maximum Likelihood trees calculated in RAxML [43] used rapid bootstrapping and searched for the best ML Tree. Bootstrapping was performed using a random seed, 100 repetitions, a general time reversible model of nucleotide substitution [50] with the I model of rate heterogeneity [51] and four discrete rate categories. Maximum Likelihood trees calculated in MrBayes [44]–[48] were set to a DNA data type, a 4×4 nucleotide model, N_st_ of 6 with a Dirichlet prior, no covarion, four states with frequencies of a Dirichlet prior, an invariable gamma (default settings), vertebrate mitochondrial code and were partitioned by codon position (1^st^, 2^nd^ or 3^rd^ base of a codon). Markov chain Monte Carlo (MCMC) was executed in two independent analyses starting from different random seeds and calculated for 1 million generations, sampling every 100 generations and performing diagnostics every 1,000 generations. Final trees were compiled from the two analyses with a burnin of 25% (15,002 total samples).

#### 2.2.1 Phylogenetic Trees Used to Determine Degree of Congruence

The phylogenies for cyt *b* and COI were assessed for their ability to show the highest degree of congruence with conventional taxonomic classifications in Super Orders, Orders, Families and genera. Although the true evolutionary history of any Class of organisms is unknown, for the purposes of comparison the following phylogenies were used. For species-level classification, Wilson and Reeder [52] (with minor modifications) was followed, so that the number of taxa that did not correctly associate with their taxonomic ranks was calculated and the percentage of correctly associated taxa was calculated. Higher level taxonomic ranks for placental mammals (Order and above) followed Murphy *et al.* [53] and marsupial Orders followed Phillips *et al.* [54] and Cardillo *et al.* [55]. For example, in the COI ML MrBayes tree four of 24 species were not associated with the other Rodentia to give a percentage correct classification of 85.7% compared with 64.3% for cyt *b*. Two values were calculated for each taxonomic group for each gene. One included all the samples that were used, whereas the second only included the putative full species. Therefore, the five samples for Asian black bear subspecies (*Ursus thibetanus* sspp.) were firstly treated as five samples for the first percentage and then recalculated as one species for the second percentage. Similar calculations were done for percentage correct assignment to Families, but inter- and intrageneric inconsistencies were noted and are commented on in the text, where applicable.

For primates, carnivorans and cetaceans (excluding artiodactyls, which are not represented as well in this study) good morphological trees or super trees (combining molecular and morphological data) are available for comparison with the molecular data from this study [2], [5], [11], [30], [56]–[66]. These allowed more detailed comparisons with those Orders, for which we have a good taxonomic representation. Significant differences from these classifications and phylogenies are discussed.

### 2.3 Data Analysis

Sequence alignments were transferred to Excel for some statistical analyses. Variation at each base position was estimated by calculating the uncertainty according to Shannon [67] adapted to aligned sequences, both DNA and amino acid, by Schneider and Stephens [68]. This value, *R_s_*, gives the degree of sequence conservation per site:
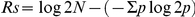
where *N* is the number of options per site and *p* is the frequency of each option per site. 100% identity at any given site for a DNA alignment would result in an *R_s_* of 2 (log_2_4 = 2 bits of information). Points of heteroplasmy were noted and included for the number of sequences, but not counted as a separate ‘base’. For example, at one base position, if out of 100 sequences 99 had an A and one had an A/G heteroplasmy, then the probability was recorded as 99% A and 0% for T, C and G.

Moving averages of identity were calculated in 101 bp, 401 bp and 601 bp sliding windows by taking a sum of the *R_s_* values and dividing it by 2*X* (2 being the *R_s_* value for 100% identity and *X* being the size of the window). Therefore, if a 101 bp sequence was 100% identical between all sequences a value of 1 would be obtained. Boxes of 401 and 601 bps were used as these are the approximate fragment sizes generally used for sequencing, when working with cyt *b* and COI, respectively. This calculation was also used to assess the level of variation over the entire genes.

MEGA 4.0 [39], [40] was used to calculate nucleotide *p*-distance; Kimura 2-parameter *p*-distance (K2P) and; synonymous and non-synonymous *p*-distances using the Nei-Gojobori method. Nucleotide *p*-distance and K2P were calculated for the first, second and third bases of each codon as well as an overall value for all bases. Synonymous and non-synonymous differences were calculated pairwise and as an overall value. Standard error estimates were calculated using 1,000 bootstrap replicates from a random seed. The data from the 236 mammal samples, 945 humans, 130 domestic cattle and 35 domestic dogs were calculated separately.

K2P values (x 100) were plotted according to their frequency. Thresholds were identified where there was a split between K2P values for within species (low values) and between species (high values). Three potential thresholds were identified. Synonymous differences were calculated per synonymous site (*d*_S_) and non-synonymous differences were calculated per non-synonymous (*d*_N_) site in both pairwise and as overall values, and *d*_S_ and *d*_N_ were plotted against each other. Owing to the large number of comparison points and the low variation within species, the intraspecific *d*_S_ and *d*_N_ were plotted as overall means with error bars representing the observed maximum and minimum values.

In addition, inter- and intraspecific data sets were combined (1,343 sequences in total – duplicate sequences were removed, one of each for dog, cattle and human) for both cyt *b* and COI to produce a single database. K2P values were calculated pairwise for all samples in the database. These values were compared to the threshold values obtained. A comparison was considered positive if it had a K2P (x100) falling below the threshold, which would indicate that the comparison was between two members of the same species. A comparison was considered negative if the K2P (x100) was greater than the threshold, which would indicate that the comparison was between two different species. A two-by-two contingency table was calculated (Table 1) where *n*_AB_ represents true positives; *n*_aB_ represents false positives; *n*_Ab_ represents false negatives; *n*_ab_ represents true negatives; *n*_A_ represents all samples/values from the same species; *n*_a_ represents all samples/values from different species; *n*_B_ represents total positive samples; and *n*_b_ represents total negative samples. Based on the contingency table (Table 1), the frequency of obtaining a false positive was estimated from the ratio of *n*_aB_ to *n*_a_ [69]. The frequency of obtaining a false negative was estimated by the ratio from *n*_Ab_ to *n*_A_ [69]. The true sensitivity (*n*_AB_/*n*_A_), specificity (*n*_ab_/*n*_a_), positive predictive value (*n*_AB_/*n*_B_) and negative predictive value (*n*_ab_/*n*_b_) were also calculated for each gene at each threshold [70].

**Table 1:** A two-by-two contingency table for K2P frequencies for the tabulation of the same species (A) or different species (a) with a K2P value (x 100) falling below or above (B or b) a threshold.

	Same species (A)	Different species (a)	Total
**< Threshold (B)**	*n*_AB_	*n*_aB_	*n*_B_
**> Threshold (b)**	*n*_Ab_	*n*_ab_	*n*_b_
**Total**	*n*_A_	*n*_a_	*n*

*n*_AB_ represents true positives; *n*_aB_ represents false positives; *n*_Ab_ represents false negatives; *n*_ab_ represents true negatives; *n*_A_ represents all samples/values from the same species; *n*_a_ represents all samples/values from different species; *n*_B_ represents total positive samples; *n*_b_ represents total negative samples and; *n* represents the total number of samples/values. Adapted from [69].

Receiver Operator Characteristic (ROC) curves were plotted using SPSS 17.0.0 (SPSS UK Ltd., Surry) and were plotted as 1–specificity (X-axis) against sensitivity (Y-axis). The ROC curves were analyzed with the smaller values indicating a positive result. Standard errors of area were calculated in a non-parametric distribution assumption with a 99% confidence level.

## Results

### 3.1 Phylogenetic Trees

Full phylogenetic trees for Maximum Parsimony, Neighbor-Joining, Minimum Evolution and Maximum Likelihood methods for the COI and cyt *b* genes can be found in the Figures S1–S11 and Tables S3 and S4. Analysis of the tree types (Table 2 and Figure 1) demonstrated that the Maximum Likelihood trees compiled using MrBayes [44]–[48] showed the highest congruence with conventional classifications and expected evolutionary history for both genes and were therefore used for detailed analysis (Figure 2).

**Figure 1:**
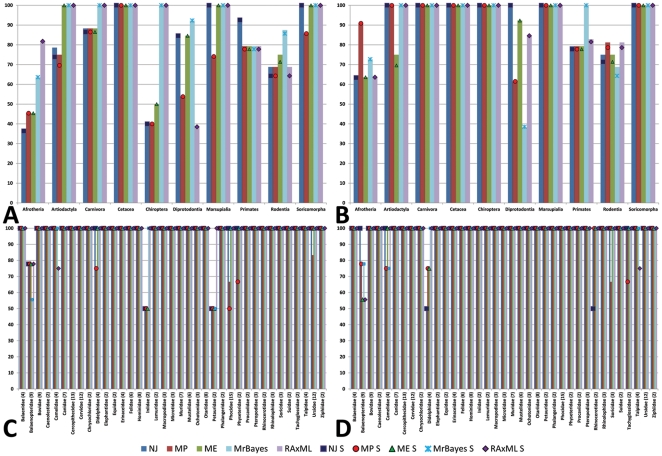
Analysis and comparison of different phylogenetic trees. An analysis of the correctly grouped Orders (a and b) and Families (c and d) for COI (a and c) and cyt *b* (b and d) from the different phylogenetic trees. Only Orders containing n≥6 were included for analysis and all Families were included for n≥2 (n is displayed in parentheses following the name of the Family). Bars indicate the percentage of correctly assigned taxonomic groups and points indicate the percentage of correctly assigned species groups within the larger taxonomic designation. NJ- Neighbor-Joining tree; MP- Maximum Parsimony tree; ME- Minimum Evolution tree; MrBayes- MrBayes Maximum Likelihood; RAxML- RAxML Maximum Likelihood; NJ S- Neighbor-Joining species placement; MP S- Maximum Parsimony species placement; ME S- Minimum Evolution species placement; MrBayes S- MrBayes Maximum Likelihood species placement and; RAxML S - RAxML Maximum Likelihood species placement.

**Figure 2:**
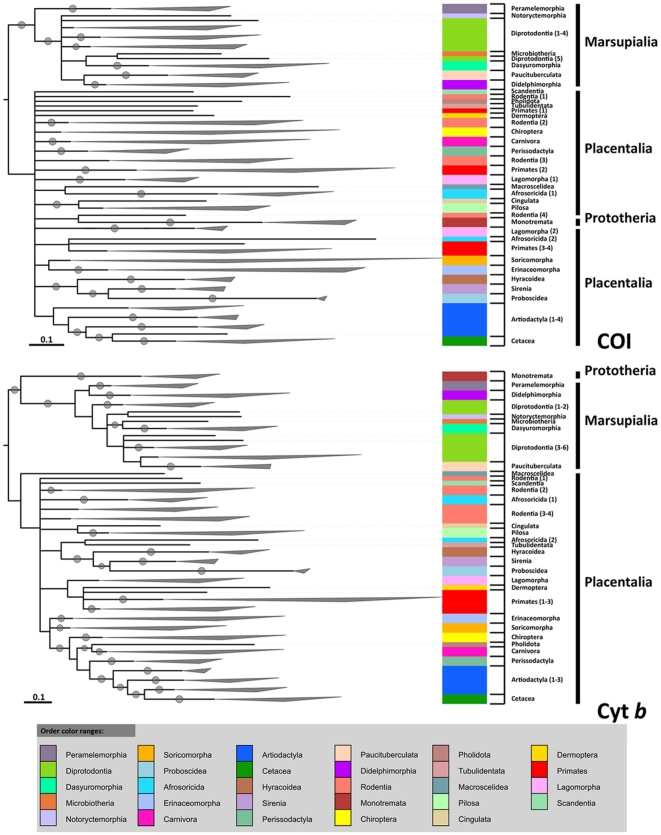
Maximum Likelihood phylogenetic trees calculated using MrBayes **[44]**–[**48**] for COI and cyt *b*. Clades have been collapsed based on Order at nodes where all subsequent branches belong to a particular Order. Full versions of these trees can be found in the Figures S3 and S8. Details of the collapsed orders can be found in Text S2.

**Table 2:** A direct comparison of the five phylogenetic trees compiled for each of COI and cyt *b*.

		Order				Family	
		n total	<100% accuracy	n chart	<100% accuracy	n total	<100% accuracy
**COI**	**Neighbor-Joining**	22	8	10	7	37	3
	**Maximum Parsimony**	22	11	10	9	37	6
	**Minimum Evolution**	22	7	10	6	37	3
	**Maximum Likelihood MrBayes**	22	5	10	4	37	2
	**Maximum Likelihood RAxML**	22	4	10	4	37	2
**Cyt** ***b***	**Neighbor-Joining**	22	3	10	3	37	1
	**Maximum Parsimony**	22	9	10	4	37	4
	**Minimum Evolution**	22	6	10	5	37	2
	**Maximum Likelihood MrBayes**	22	3	10	3	37	2
	**Maximum Likelihood RAxML**	22	4	10	4	37	2

n total refers to the total number of groups (Orders or Families) present in the trees. n chart refers to only those Orders which were used in Figure 1. <100% accuracy refers to any grouping which did not correspond to the expected grouping as given by conventional morphological and taxonomic analysis as described in Materials and Methods section 2.2.1. The MrBayes Maximum Likelihood trees show the most accuracy overall. The Maximum Parsimony trees showed the least accuracy for both genes at both the Order and Family levels.

### 3.2 Data analysis

Sequence data, both interspecific and intraspecific, were analyzed and results are summarized in Table 3.

**Table 3:** A comparison of the intra- and inter-specific variation in cyt *b* and COI genes.

	INTERSPECIFIC VARIATION		INTRASPECIFIC VARIATION					
	Mammalian samples (n = 236)		*Homo sapiens* (n = 945)		*Bos taurus* (n = 130)		*Canis familiaris* (n = 35)	
	Cyt *b*	CO1	Cyt *b*	CO1	Cyt *b*	CO1	Cyt *b*	CO1
**Size (bp)**	1130–149	1537–1557	1141	1542	1140	1545	1140	1545
**Heteroplasmy**	0	0	7	4	0	0	0	1
**Total variable sites**	892	877	206	191	36	39	18	28
**in a single sample**	318	198	101	81	23	31	8	6
***p*** **-distance (x100)**								
**1^st^ base**	16.876±1.067	08.662±0.690	0.265±0.077	0.123±0.063	0.120±0.048	0.015±0.007	0.188±0.097	0.086±0.045
**2^nd^ base**	06.218±0.645	01.768±0.287	0.135±0.074	0.017±0.008	0.024±0.012	0.027±0.012	0.015±0.015	0.000
**3^rd^ base**	47.775±0.808	51.525±0.666	0.614±0.151	0.602±0.127	0.097±0.024	0.174±0.058	0.552±0.186	0.733±0.180
**Mean**	23.602±0.662	20.644±0.474	0.338±0.065	0.247±0.049	0.080±0.019	0.072±0.020	0.252±0.075	0.273±0.064
**Maximum**	32.651	26.090	1.150	1.040	0.526	0.518	0.789	0.777
**Minimum**	0.000	0.000	0.000	0.000	0.000	0.000	0.000	0.000
**Kimura 2-parameter ** ***p*** **-distance (x100)**								
**1^st^ base**	19.485±1.392	09.363±0.823	0.267±0.079	0.124±0.064	0.121±0.049	0.015±0.006	0.189±0.100	0.086±0.045
**2^nd^ base**	06.588±0.721	01.797±0.293	0.136±0.075	0.017±0.008	0.024±0.012	0.027±0.012	0.015±0.014	0.000
**3^rd^ base**	80.217±2.398	97.677±2.474	0.620±0.159	0.607±0.124	0.097±0.025	0.175±0.060	0.557±0.197	0.741±0.191
**Mean**	28.794±1.007	24.540±0.753	0.339±0.066	0.248±0.049	0.081±0.019	0.072±0.020	0.253±0.076	0.274±0.064
**Maximum**	43.648	32.605	1.160	1.050	0.529	0.520	0.795	0.783
**Minimum**	0.000	0.000	0.000	0.000	0.000	0.000	0.000	0.000
**Synonymous and Non-synonymous ** ***p*** **-distance Nei-Gojobori**								
**S (x100)**	63.309±0.971	71.264±0.881	0.907±0.213	0.811±0.163	0.198±0.072	0.244±0.080	0.930±0.292	1.075±0.239
**N (x100)**	10.497±0.769	03.976±0.384	0.152±0.047	0.059±0.029	0.041±0.010	0.016±0.006	0.033±0.016	0.014±0.010

n =  sample size. Standard errors were calculated using 1,000 bootstrap repetitions. 1^st^, 2^nd^ and 3^rd^ base refer to the position within a codon.

**Figure 3:**
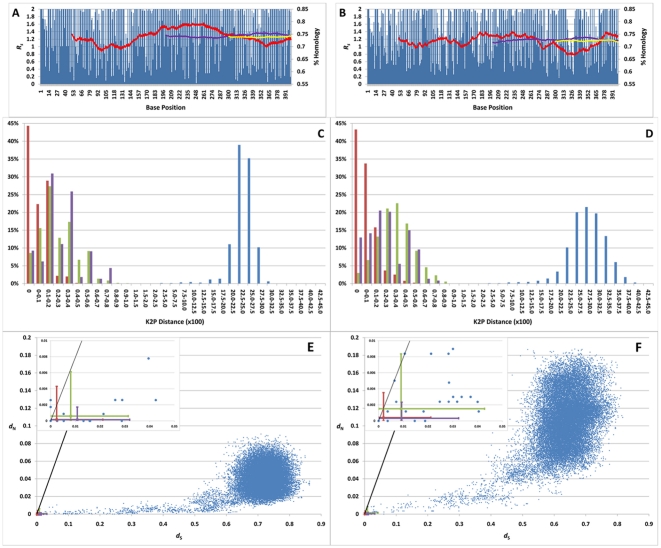
A comparison of the COI and cyt *b* genes. a and b) The *R_s_* values for the first 400 base pairs of the COI (a) and cyt *b* (b) genes. Sliding windows of identity are shown for blocks of 101 bp (red), 401 bp (purple) and 601 bp (yellow), and are shown as a percentage of completely conserved sequences. Values for entire genes can be found in Figure S12. c and d) A histogram of the Kimura 2-parameter *p*-distances for COI (c) and cyt *b* (d) for pairwise comparisons within species for domestic cattle (red), domestic dogs (purple), humans (green) and between other mammalian species (blue). e and f) The synonymous differences per synonymous site (*d*_S_) versus the nonsynonymous differences per nonsynonymous site (*d*_N_) for COI (e) and cyt *b* (f) calculated in pairwise fashion within species for cattle (red), dogs (purple), humans (green) and between other mammalian species (blue). Within-species comparisons are shown in the inlays and are displayed as a mean value with error bars representing maxima and minima. The reference line indicates *d*_S_ = *d*_N_.

Variability (*R_s_*) was determined at each base position for each gene (Figure 3a, b and Figure S12). Between-species COI showed an average *R_s_* value of 74.5% of a completely conserved gene. Of 1,557 base pairs (bp) (largest variant), 56.3% were variable in at least one sample. Sliding windows within COI of 101, 401 and 601 bp showed average *R_s_* values of 74.6, 75.2 and 75.4% of a conserved sequence, respectively. Cyt *b* showed an average *R_s_* value of 69.9% of a completely conserved gene. Of the total 1,149 bp of cyt *b*, 77.6% showed variation in at least one sample. Sliding windows within cyt *b* of 101, 401 and 601 bp gave average *R_s_* values of 70.2, 70.6 and 70.6% of a conserved sequence, respectively.

**Figure 4:**
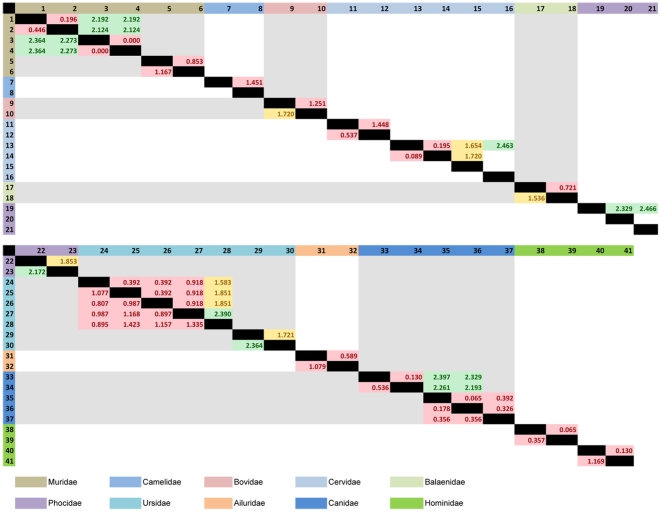
The Kimura 2-parameter *p*-distance (x100) for COI in the upper diagonal and cyt *b* in the lower diagonal. Only values less than 2.5 are shown and all other comparisons showed values above 2.5. Red shading indicates <1.5, yellow shading indicates 1.5<>2 and green shading indicates 2<>2.5. Species included are: *Mus musculus musculus* (1); *M. m. molossinus* (2); *M. musculus* (3); *M. m. domesticus* (4); *Rattus norvegicus* (Wistar) (5); *R. norvegicus* (BN/SsNHsdMCW) (6); *Camelus ferus* (7); *C. bactrianus* (8); *Bos indicus* (9); *B. taurus* (10); *Muntiacus reevesi micrurus* (11); *M. reevesi* (12); *Cervus nippon centralis* (13); *C. n. yesoensis* (14); *C. n. yakushimae* (15); *C. n. taiouanus* (16); *Eubalaena japonica* (17); *E. australis* (18); *Pusa sibirica* (19); *P. caspica* (20); *P. hispida* (21); *Phoca vitulina* (22); *Phoca largha* (23); *Ursus thibetanus ussuricus* (24); *U. thibetanus* (25); *U. t. thibetanus* (26); *U. t. formosanus* (27); *U. t. mupinensis* (28); *U. maritimus* (29); *U. arctos* (30); *Ailurus fulgens* (31); *A. f. styani* (32); *Canis lupus chanco* (33); *C. l. laniger* (34); *C. l. lupus* (35); *C. lupus* (36); *C. familiaris* (37); *Homo sapiens* (38, 39); *Gorilla gorilla gorilla* (40) and; *G. gorilla* (41). The colored headings represent different Families.

Average K2P values (x 100) were plotted according to frequency when the four data sets were analyzed independently (Figure 3c, d). There is a gap between intraspecific and interspecific K2P values, ranging between 1.5 and 2.5 for both genes. Intraspecific K2P values were <1.5 with maximum values of 1.05 and 1.16 for COI and cyt *b*, respectively. The highest intraspecific value was between two human cyt *b* samples. No intraspecific K2P *p*-distance comparison was >1.5 and few inter-specific values were <2.5 (Figure 4). This suggested three potential K2P (x 100) threshold values of 2.5, 2.0 and 1.5, which would distinguish intra- and interspecific differences.

When subspecific and intraspecific comparisons were removed, the average between-species K2P (x 100) values were 24.6±2.9 and 28.8±4.8 for COI and cyt *b*, respectively. Within-Order K2P comparisons (for Orders with n≥3) showed average values of 20.2±2.8 and 22.4±4.0 for COI and cyt *b*, respectively. Within-Order values were greater in cyt *b* in all Orders by, on average, 2.35 except in the Lagomorpha where the COI average was greater by 0.27. Average intraspecific K2P (x 100) values for human, domestic cattle and domestic dog samples were: 0.25±0.18 (COI) and 0.34±0.16 (cyt *b*); 0.07±0.08 (COI) and 0.08±0.09 (cyt *b*) and; 0.27±0.18 (COI) and 0.25±0.18 (cyt *b*), respectively.

Pairwise comparisons of synonymous (*d_S_*) and non-synonymous substitution rates (*d_N_*) were plotted against each other (Figure 3e, f). Between-species COI showed a greater average synonymous substitution rate (0.7126±0.0686) than cyt *b* (0.6331±0.0701). However, cyt *b* showed a greater average non-synonymous substitution rate than COI (0.1049±0.0311 and 0.0398±0.0148, respectively). For COI two pairs of samples showed a *d_N_*>*d_S_* (*Gorilla gorilla* and *G. g. gorilla*, and *Cervus nippon centralis* and *C. n. yesoensis*); all cyt *b* comparisons showed *d_N_*≤*d_S_*.

Combined-data-set K2P pairwise comparisons (901,153 comparisons) showed similar results to those when analysed separately. All *Bos taurus* and *B. indicus* comparisons fell below 1.5 in COI, but only two fell below 1.5 in cyt *b* with the rest falling between 1.5 and 2. All samples of *Canis familiaris*, compared with *C. lupus* and *C. l. lupus*, fell below 1.5 for both COI and cyt *b*, but seven of the *C. familiaris* samples showed 100% sequence match (K2P = 0) with the *C. l. lupus* sample in COI. Also within COI 30 of the *C. familiaris* samples showed K2P values between 2 and 2.5, when compared to *C. l. laniger* and 16 K2P values were between 2 and 2.5 when compared with *C. l. chanco*.

A K2P threshold of 1.5 showed that for COI the false positive rate was 4.85×10^−4^ and the positive predictive value was 0.9995. For cyt *b* the false positive rate was 2.02×10^−4^ and the positive predictive value was 0.9998. Values for comparisons of sensitivity and specificity for a threshold of 1.5, along with results for thresholds of 2.0 and 2.5, are found in Table 4. Results for the ROC curves can be found in the Figure S13 and Table S5. No differences are evident between the ROC curves for the two genes.

**Table 4:** Results of the analyses from the two-by-two contingency table.

	Cyt *b*	COI		Cyt *b*	COI
**Total greater than (** ***n*** **_b_)**			**Rate of False Negative (** ***n*** **_Ab_/** ***n*** **_A_)**		
2.5%	444962	444906	at 2.5%	0	0
2.0%	444968	444964	at 2.0%	0	0
1.5%	445097	444971	at 1.5%	0	0
**Total less than (** ***n*** **_B_)**			**Sensitivity (** ***n*** **_AB_/** ***n*** **_A_)**		
2.5%	456191	456247	at 2.5%	1	1
2.0%	456185	456189	at 2.0%	1	1
1.5%	456056	456182	at 1.5%	1	1
**False Negative (** ***n*** **_Ab_)**			**Specificity (** ***n*** **_ab_/** ***n*** **_a_)**		
at 2.5%	0	0	at 2.5%	0.999495	0.999369
at 2.0%	0	0	at 2.0%	0.999508	0.999499
at 1.5%	0	0	at 1.5%	0.999798	0.999515
**False Positive (** ***n*** **_aB_)**			**Positive Predictive Value (** ***n*** **_AB_/** ***n*** **_B_)**		
at 2.5%	225	281	at 2.5%	0.999507	0.999384
at 2.0%	219	223	at 2.0%	0.999520	0.999511
at 1.5%	90	216	at 1.5%	0.999803	0.999527
**Rate of False Positive (** ***n*** **_aB_/** ***n*** **_a_)**			**Negative Predictive Value (** ***n*** **_ab_/** ***n*** **_b_)**		
at 2.5%	0.000505	0.000631	at 2.5%	1	1
at 2.0%	0.000492	0.000501	at 2.0%	1	1
at 1.5%	0.000202	0.000485	at 1.5%	1	1

Thresholds were set at K2P values (x 100%) of 2.5, 2.0 and 1.5% such that the negative predictive value (the probability that a comparison will be from two different species) was 1.

## Discussion

### 4.1 Accuracy of the Phylogenetic Trees

Analysis of the phylogenetic trees showed that overall the Maximum Likelihood phylogenetic trees constructed in MrBayes show the highest congruences with conventional taxonomic groupings. The ML trees show the least number of inconsistencies when compared to traditional morphological and other molecular studies based on different gene loci, and taxonomic classifications and expected relationships between species. This method was used in further analyses in this study.

Although cyt b showed a very high congruence (95.85%) with conventional classifications at Super Order, COI was very poor (78.34%) (Figure 2, un-collapsed trees can be found in Figures S3 and S8). COI grouped all species correctly in Xenarthra, but members of Afrotheria were split, with only 63.6% (elephants, tenrec, sirenians and hyraxes) associated together. Cyt *b* also correctly grouped all xenarthrans together, but gave a better grouping of Afrotherians (72.7%), with only the golden moles and elephant shrew grouped incorrectly. The results for Euarchontoglires and Laurasiatheria were very different for each gene. COI was much less able to group members of the Euarchontoglires (63.0%) and Laurasiatheria (79.4%) together, whereas cyt *b* performed much better with 87.0% of Euarchontoglires and 100% of Laurasiatheria grouped correctly together, so that overall cyt *b* correctly placed more of the 217 mammal species in the correct Super Order than COI. Both genes correctly assigned marsupials and monotremes to their respective groups. However, relationships between Super Orders were difficult to interpret, owing to a lack of resolution of the branching of Super Orders. For cyt *b* most Afrotherians were closest to the Xenarthra, which is regarded as basal among placentals (e.g. [1], [10], [71]), although not in all phylogenies (e.g. [72]) as found here. The high degree of splitting among members of the Laurasiatheria and Euarchontoglires for COI makes further interpretation difficult. The monotremes grouped together correctly for both genes; for cyt *b* they were the sister group to marsupials and basal to a mixed grouping of Afrotherians, rodents and xenarthrans, but for COI the Eurasian red squirrel, *Sciurus vulgaris*, was a sister species to the monotremes between the perissodactyls and Afrotherians.

One notable aspect of these phylogenetic reconstructions is the difference in branch lengths for different mammal groups. Larger species, such as carnivorans, cetaceans, artiodactyls, primates, etc., tended to have much shorter branch lengths than for smaller species, such as bats, rodents, hedgehogs, shrews, marsupials and monotremes. This has been noted in previous phylogenetic reconstructions and different explanations have been proposed, including differences in generation times and longevity, but recent analyses suggest that mutation rates differ for mtDNA between different mammal lineages [73]. These differences would significantly affect phylogenetic reconstruction, owing to the distortion of relationships between otherwise closely related taxonomic groups. However, this is only understood for a limited number of species.

At the level of Order cyt *b* grouped correctly 94.31% of 211 mammal species and COI gave a slightly lower percentage of 93.36%. For COI six placental mammal Orders were split with maximum correct groupings ranging from 66.7% to 85.7%, and one marsupial Order (Diprotodontia) was split with 92.3% correct membership. In contrast, for cyt *b* only two placental Orders (Lagomorpha and Rodentia) did not correctly group species, varying between 60.0 and 64.3% correct membership, and one marsupial Order (Diprotodontia) had one split, giving a correct grouping of 61.5%. However, splitting of Orders was mostly caused by particular species associating oddly with unrelated groups. For example, for cyt *b* the rodents were split into three groups by Afrotherians, xenarthrans and the tree shrew, *Tupaia belangeri*. For COI the association pattern was far less clear; rodents were split into four groups with fat dormouse, *Glis glis*, Eurasian red squirrel and guinea pig, *Cavia porcellus*/cane rat, *Thryonomys swinderianus*, separated from the main group, Horsfield' tarsier, *Tarsius spectrum*, colugo, *Galeopterus variegatus*, aardvark, *Orycteropus afer*, and pangolin, *Manis longicaudatus*, are associated with each other, the prosimian primates are separated from the remaining primates, and the lagomorphs are divided by the elephant shrew, *Macroscelideus proboscideus*, and golden moles.

At the level of Family cyt *b* correctly grouped 98.16% of 163 mammal species compared with 96.93% for COI. For COI, one placental Family (Balaenopteridae) only correctly grouped 55.6% of species, while one marsupial Family (Petauridae) was split for its two species. For cyt *b*, the Camelidae showed only 75% correct grouping and the Balaenopteridae showed 77.8% correct association of its species. However, it should be noted that with both genes, the gray whale's (*Eschrichtius robustus*, Family Eschrichtidae) placement with respect to the Balaenopteridae makes the latter paraphyletic, although this has been recorded previously [11], [30], [64], [66], indicating a significant morphological divergence of the gray whale from a balanaeopterid ancestor [62], [66]. However, other recent phylogenetic studies of mysticetes place it basal to the Balaenopteridae [60]–[62], [65]. Not surprisingly, as potential group sizes fall from Super Order through Order to Family, so the chances of species misclassification reduce, but cyt *b* shows congruences of >94% for all taxonomic ranks compared with conventional classifications and phylogenies.

At generic and intrafamilial phylogenetic levels, most relationships were reconstructed by cyt *b* as expected and are congruent with a recent molecular supertree for the Carnivora [59]. Exceptions were among phocine seals, where relationships between three genera (*Phoca*, *Pusa* and *Halichoerus*) were somewhat anomalous, with the Baikal seal, *Pusa sibirica*, not correctly grouped with other *Pusa* spp., relative to the grey seal, *Halichoerus grypus*, but similar results were obtained by Arnason *et al.* [74]. Hooker's sealion, *Phocarctos hookeri*, and Australian sealion, *Neophoca cinerea*, were placed within the fur seals, *Arctocephalus* spp., making the latter paraphyletic, but similar paraphyly was recorded by Agnarsson *et al.* [59]. Cyt *b* placed the colobus monkeys within an expected langur and odd-nosed colobines clade, thus isolating the entellus langur, *Semnopithecus entellus*, but for all other primates phylogenetic relationships were reconstructed as expected [37], [58]. MtDNA has produced anomalous phylogenies for langurs in other studies, which was interpreted as being due to ancient hybridisation events [75], [76]. The red deer, *Cervus elaphus*, was shown as a sister species to the Formosan sambar, *Rucervus unicolor*, rather than the expected sister grouping with sika deer, *C. nippon* sspp. [63]. The humpback whale, *Megaptera novaeangliae*, is a sister species to the fin whale, *Balaenoptera physalus*, but similar results have been recorded in other recent phylogenetic studies of cetaceans [60]–[62], [65], [66], suggesting that taxonomic relationships require further investigation; ancient hybridisation has been ruled out as influencing baleen whale phylogenetics [66]. The gray whale, *Eschrichtius robustus* (Family Eschrichtidae), is the sister species to the humpback/fin whale clade rather than basal to the Balaenopteridae as in recent molecular and morphological/molecular phylogenies [60]–[62], [65], [66]. There was no expected sister grouping of goat, *Capra hircus*, and sheep, *Ovis aries*, although other relationships within the ovicaprines were broadly as expected [11], [30]. Among marsupials, the anomalous banded hare wallaby, *Lagostrophus fasciatus*, was expected to be divergent from the rufous hare wallaby, *Lagorchestes hirsutus*, and wallaroo, *Macropus robustus*, as shown by cyt *b* [55].

For COI generic and intrafamilial relationships were almost as good and in some cases provided better phylogenetic reconstructions than for cyt *b*. The apparently anomalous position of the gray whale and the humpback whale among *Balaenoptera* spp., was repeated, albeit with a different pattern of relationships, and the gray seal formed a single clade with the *Pusa* spp. seals. However, an expected sister grouping of *Phocarctos hookeri* and *Neophoca cinerea* maintained the paraphyly of *Arctocephalus* spp. The Formosan sambar, *Rucervus unicolor*, occurred in the same clade as *Cervus* spp., but the relationship between red and sika deer was reconstructed as expected [63]. The Asian colobine phylogeny was reconstructed as expected based on morphology, with a sister grouping of the langurs and the odd-nosed colobines.

### 4.2 Sequence Analysis

Comparison of sequence alignments showed cyt *b* as more variable for both inter- and intraspecific comparisons, which is in line with previous studies [31]. Estimates of substitutions per site, using the *p*-distance and K2P distance (Table 3), showed greater substitution rates at the third nucleotide position followed by the first and then the second, as expected in coding genes, owing to redundancy in the genetic code. An exception was the sample of 130 *Bos taurus* individuals, which showed greater substitution rates at the first nucleotide position for cyt *b* and at the second position for COI.

Sequence analysis also showed that cyt *b* contains 21.3% more base positions (relative to the size of the gene, largest variant) that are variable than does COI. *R_s_* values demonstrated more variability (+3.1%) than COI in a sequence that is 408 bp shorter (1149 bp for cyt *b* and 1557 bp for COI, largest variants). For both genes within the 945 human samples ≈50% of the variable sites were variable in only one sample (Table 3) with similar results between the 130 domestic cattle individuals. Domestic dog samples showed a different pattern in both genes with less mutations appearing in only one sample.

The average number of synonymous substitutions per synonymous site was greater in both genes than the average number of nonsynonymous substitutions per nonsynonymous site for all data sets. Pairwise comparisons showed *d_S_>d_N_* and are almost equal between the two genes, indicating that both genes are in states of purifying and neutral selection. Two COI pairwise comparisons showed *d_N_>d_S_*; *Gorilla gorilla* and *G. g. gorilla*, and *Cervus nippon centralis* and *C. n. yesoensis*. However, these two comparisons showed a *d_N_* almost equal to *d_S_*, and almost on the reference line (Figure 3e inlay). They are within the *d_N_* and *d_S_* values obtained for domestic cattle and human intraspecific comparisons.

### 4.3 Determining Thresholds

Based on the K2P histogram (Figure 3), a clear separation between inter- and intraspecific comparisons can be seen, falling between 1.5 and 2.5. This is similar to what has been identified in similar studies with other Classes of organisms [22], [33], [77]–[81]. With all intraspecific comparisons falling well below K2P  = 1.5, the largest being 1.16, our analysis suggests that a K2P  = 1.5 can be adopted as a minimum threshold for mammalian species separation. A K2P <1.5 indicates the samples come from the same species and a K2P>2.5 indicates that the sample comes from a different mammalian species for COI and cyt *b*. This leaves a gray area of 1.5≤K2P≤2.5, where there is the possibility that intraspecific variation is greater than predicted and would require analysis of more than one locus. We feel that this gray area is important because, despite the huge number of sequences on the databases, there are still insufficient data to properly assess intraspecific variability for all organisms. These thresholds can be refined as more intraspecific analyses are undertaken.

### 4.4 Inter- and Intra-specific Analyses

The K2P distance [82] is the most widely used metric in analyses using COI [83] and was used for our comparisons of inter- and intraspecific differences. Using 217 species (236 samples) to examine interspecific variation, cyt *b* demonstrated greater K2P values (x100) with a greater standard deviation at both the species (average 28.8±4.8) and Order (average 22.4±4.0) levels compared with those from COI (24.6±2.9 and 20.2±2.8 on average for species and Order, respectively).

Interspecific analysis showed that most (>99.99%) interspecific comparisons fell above K2P  = 2.5 (27 and 37 out of 27,727 comparisons for cyt *b* and COI, respectively, fell below). Those comparisons falling below a K2P distance (x100) of 2.5 were generally between species (or some subspecies) or genera. COI K2P values showed two intrageneric comparisons with values <1.5 (1.251 between *Bos indicus* and *B. taurus*; 0.721 between *Eubalaena japonica* and *E. australis*), but cyt *b* K2P values for these two comparisons were >1.5 (1.720 between *B. indicus* and *B. taurus*; 1.536 between *E. japonica* and *E. australis*). All cyt *b* K2P values <1.5 were between subspecies (Figure 4). However, the different levels of K2P values for COI and cyt *b* reflect taxonomic uncertainty as to how these taxon pairs are treated; in some cases they are full species, and in others they are regarded as conspecific with *B. taurus* and *E. australis* respectively.

One comparison gave K2P  = 0 for both COI and cyt *b* between *Mus musculus* and *M. m. domesticus*, which was almost certainly because of inaccurate taxonomic designation of the *M. musculus* sample, which is likely to be *M. m. domesticus* (N.B. The subspecies *musculus* and *domesticus* are often treated as separate species). This misclassification is a recognized problem associated with online databases [84], [85]. Conversely, all Palaearctic badgers were treated as a single species, *Meles meles*, until a recent morphological analysis revealed three species, including the Japanese badger, *M. anakuma* [86], which is confirmed by K2P values of 8.736 for cyt *b* and 6.734 for COI in this study.

Intraspecific variation was examined using mtDNA data from 945 human, 35 domestic dog and 130 domestic cattle samples. For both genes and all three data sets, intraspecific variation was below a K2P value (x100) of 1.5. This is similar to what was found in a similar, but smaller study [81]. Comparisons of the two genes showed that cyt *b* demonstrated greater intraspecific K2P values for human and domestic cattle samples (1.367 and 1.125 times greater than average COI values, respectively). However, domestic dog samples showed average K2P values 1.083 times greater in the COI comparison. The amalgamation of all four data sets showed all *Bos taurus* and *B. indicus* comparisons falling below K2P  = 1.5 in COI, but only two fell below 1.5 in cyt *b*, with the rest falling between 1.5 and 2. This could be due to misclassification of some of the *B. indicus* samples (which may have been *B. taurus*) or might be due to potential hybrid individuals.

Conversely, even though COI showed greater intraspecific variation within the domestic dog samples, it did not perform as well as cyt *b* in the combined data set. Seven of the *Canis familiaris* samples showed 100% sequence match (K2P  = 0) with the *C. l. lupus* sample in COI. The closest sequence match between *C. familiaris* and *C. l. lupus* in cyt *b* was K2P (x100)  = 0.09 for one sample. *C. lupus* from the Middle East is known to be the ancestor of domestic dogs, perhaps with various genetic infusions from local northern wolf populations [87]. Also within COI, 30 of the *C. familiaris* samples showed K2P values between 2 and 2.5, when compared to *C. l. laniger* and 16 domestic dog samples had K2P values between 2 and 2.5 when compared with *C. l. chanco*. This was not observed within the cyt *b* comparison. This indicates that cyt *b* is better suited for species differentiation, being able to better separate closely related congeneric species. However, it should be noted that recent molecular studies have suggested that *C. lupus* may well represent more than one species, with Himalayan/Tibetan wolves, *C. l. chanco/laniger*, regarded as possibly specifically distinct from *C. lupus* [88].

Other anomalies within the combined dataset K2P comparisons are: *Camelus bactrianus* and *C. b. ferus* show K2P <1.5 with COI but >2.5 with cyt *b* and *Ursus thibetanus mupinensis* with *U. t. ussuricus*, *U. thibetanus*, *U. t. thibetanus* and *U. t. formosanus* show K2P >1.5 with COI but <1.5 for cyt *b*. Domestic and wild Bactrian camels are morphologically similar, but it is increasingly normal for wild counterparts for domestic mammals to be treated as distinct species, following the ruling of the International Commission for Zoological Nomenclature [89]. A recent mtDNA study supports the specific differentiation between wild and domestic Bactrian camels [90]. The intraspecific taxonomy of the Asian black bear is in much need of revision [91], but only *U. t. ussuricus* and island populations (e.g. *U. t. formosanus*) are likely to be regarded as subspecifically distinctive, owing to geographical isolation. In both cases *cyt b* is likely to be giving a more accurate assessment of the taxonomic distinctiveness of these taxa.

### 4.5 Statistical Analysis

Statistical tests have been applied, for the first time to our knowledge, to sequence comparisons for identification purposes. Although the statistical results are similar for both genes (ROC curves show no differences, see Figure S13 and Table S5), cyt *b* displays a greater ability to distinguish between samples originating from the same or different species.

All potential threshold values showed no false negatives for both genes, meaning that none of the intraspecific comparisons in our study would be shown to originate from two different species. The two genes do start to be differentiated by the rate of false positives, where COI has a value (at a threshold of 1.5) 2.4 times great than cyt *b*. This is shown in the false positives obtained; 90 for cyt *b* versus 216 for COI. These values become almost equal if the threshold is increased to 2.0, but an increase of the threshold also decreases the positive predictive value. This demonstrates that a K2P value of 1.5 provides the most accurate threshold value to determine the specific identity of unknown samples.

This is the first report that applies a statistical approach to determine the accuracy of sequence data being used for identification purposes; the key threshold value of 1.5 for cyt *b* will now allow unknown samples to be identified with confidence when compared to database or reference samples. This method sets a precedent that can be applied for use with other online sequence databases. These methods can also be applied to other studies or for other genes, for validation purposes.

### 4.6 Conclusions

Although our comparisons for COI and cyt *b* show similar results, cyt *b* demonstrates: (i) greater congruence with conventional mammalian phylogeny; (ii) greater variation in base pairs in a shorter sequence; (iii) that its intraspecific variation is similar to that of COI and still remains below a nominal threshold and; (iv) that it has a rate of false positive less than half that of COI and a greater positive predictor value. This is the first study to compare the relative values of cyt *b* and COI for phylogenetic reconstruction and identification of mammalian species despite much investment in the previous use of both these loci. For the first time statistical confidence has been applied to species identification. If one locus is to be used as a standard for mammalian species phylogeny and identification, our data supports the use of cyt *b* over that of COI.

## Supporting Information

Text S1 Supplementary references.
(0.05 MB DOC)

Text S2 Supplementary Figure 2 caption.
(0.03 MB DOC)

Figure S1 The evolutionary history inferred using the Minimum Evolution method [S115] for COI. The optimal tree with the sum of branch length  = 17.26235975 is shown. The percentage of replicate trees in which the associated taxa clustered together in the bootstrap test (1,000 replicates) [S116] are show as symbols on the branches (for values >75%). The tree is drawn to scale, with branch lengths in the same units as those of the evolutionary distances used to infer the phylogenetic tree. The evolutionary distances were computed using the Kimura 2-parameter method [S117] and are in the units of the number of base substitutions per site. The ME tree was searched using the Close-Neighbor-Interchange (CNI) algorithm [S118] at a search level of 1. The Neighbor-joining algorithm [S119] was used to generate the initial tree.
(10.30 MB EPS)

Figure S2 The evolutionary history inferred using the Maximum Parsimony method [S120] for COI. The consensus tree inferred from 15 most parsimonious trees is shown. The consistency index is 0.051612, the retention index is (0.500358), and the composite index is 0.027801 (0.025824) for all sites and parsimony-informative sites (in parentheses). The percentage of parsimonious trees in which the associated taxa clustered together are shown as symbols (for values >75%). The MP tree was obtained using the Close-Neighbor-Interchange algorithm [S118] with search level 3 [S110–18] in which the initial trees were obtained with the random addition of sequences (10 replicates). There were a total of 1537 positions in the final dataset, out of which 744 were parsimony informative.
(10.29 MB EPS)

Figure S3 The evolutionary history inferred using the Maximum Likelihood method as calculated using MrBayes [S111–112] for COI. Markov chain Monte Carlo (MCMC) [S121] was executed in two independent analyses starting from different random seeds, parameters were DNA data type, a 4×4 nucleotide model, Nst of 6 with a Dirichlet prior, no covarion, four states with frequencies of a Dirichlet prior, an invariable gamma (default settings), vertebrate mitochondrial code and were partitioned by codon position (1st, 2nd or 3rd base of a codon) [S122–123]. The consensus tree was inferred from 15,002 trees. Total tree length is 59.710509 with variance of 2.341455. The median tree length of all sampled trees is 59.749; the lower and upper boundaries of the 95% credibility interval are 56.876 and 62.723, respectively. The six reversible substitution rates, four stationary state frequencies (pi), the shape of the gamma distribution (α) and the proportion of invariable sites (pinvar) can be found in Table S3. Posterior probabilities are shown as symbols on the branches (for values >75%).
(10.28 MB EPS)

Figure S4 The evolutionary history inferred using the Neighbor-Joining method [S119] for COI. The optimal tree with the sum of branch length  = 17.29940543 is shown. The percentage of replicate trees in which the associated taxa clustered together in the bootstrap test (1,000 replicates) [S116] are show as symbols on the branches (for values >75%). The tree is drawn to scale, with branch lengths in the same units as those of the evolutionary distances used to infer the phylogenetic tree. The evolutionary distances were computed using the Kimura 2-parameter method [S117] and are in the units of the number of base substitutions per site.
(10.29 MB EPS)

Figure S5 The evolutionary history inferred using the Maximum Likelihood method as calculated using RAxML [S113] for COI. Rapid bootstrapping was used followed by searching for the best ML Tree. Bootstrapping was performed using a random seed, 100 repetitions, a general time reversible model of nucleotide substitution [S124] with the I model of rate heterogeneity [S125] and four discrete rate categories. Tree length is 52.96548. The six reversible substitution rates, four stationary state frequencies (pi), the shape of the gamma distribution (α) and the proportion of invariable sites (pinvar) can be found in Table S4. Posterior probabilities are shown as symbols on the branches (for values >75%).
(10.27 MB EPS)

Figure S6 The evolutionary history inferred using the Minimum Evolution method [S115] for cyt *b*. The optimal tree with the sum of branch length  = 19.25186677 is shown. The percentage of replicate trees in which the associated taxa clustered together in the bootstrap test (1,000 replicates) [S116] are shown as symbols on the branches (for values >75%). The tree is drawn to scale, with branch lengths in the same units as those of the evolutionary distances used to infer the phylogenetic tree. The evolutionary distances were computed using the Kimura 2-parameter method [S117] and are in the units of the number of base substitutions per site. The ME tree was searched using the Close-Neighbor-Interchange (CNI) algorithm [S118] at a search level of 1. The Neighbor-joining algorithm [S119] was used to generate the initial tree.
(10.27 MB EPS)

Figure S7 The evolutionary history inferred using the Maximum Parsimony method [S120] for cyt *b*. The consensus tree inferred from 15 most parsimonious trees is shown. The consistency index is 0.065208, the retention index is (0.508168), and the composite index is 0.034876 (0.033137) for all sites and parsimony-informative sites (in parentheses). The percentage of parsimonious trees in which the associated taxa clustered together are shown as symbols (for values >75%). The MP tree was obtained using the Close-Neighbor-Interchange algorithm [S118] with search level 3 [S110–118] in which the initial trees were obtained with the random addition of sequences (10 replicates). There were a total of 1124 positions in the final dataset, out of which 710 were parsimony informative.
(10.27 MB EPS)

Figure S8 The evolutionary history inferred using the Maximum Likelihood method as calculated using MrBayes [S111–112] for cyt *b*. Markov chain Monte Carlo (MCMC) [S121] was executed in two independent analyses starting from different random seeds, parameters were DNA data type, a 4×4 nucleotide model, Nst of 6 with a Dirichlet prior, no covarion, four states with frequencies of a Dirichlet prior, an invariable gamma (default settings), vertebrate mitochondrial code and were partitioned by codon position (1st, 2nd or 3rd base of a codon) [S122–123]. The consensus tree was inferred from 15,002 trees. Total tree length is 59.66446 with variance of 2.882398. The median tree length of all sampled trees is 59.532; the lower and upper boundaries of the 95% credibility interval are 56.701 and 63.177, respectively. The six reversible substitution rates, four stationary state frequencies (pi), the shape of the gamma distribution (α) and the proportion of invariable sites (pinvar) can be found in Table S3. Posterior probabilities are shown as symbols on the branches (for values >75%).
(10.28 MB EPS)

Figure S9 The evolutionary history inferred using the Neighbor-Joining method [S119] for cyt *b*. The optimal tree with the sum of branch length  = 19.31726074 is shown. The percentage of replicate trees in which the associated taxa clustered together in the bootstrap test (1,000 replicates) [S116] are shown as symbols on the branches (for values >75%). The tree is drawn to scale, with branch lengths in the same units as those of the evolutionary distances used to infer the phylogenetic tree. The evolutionary distances were computed using the Kimura 2-parameter method [S117] and are in the units of the number of base substitutions per site.
(10.27 MB EPS)

Figure S10 The evolutionary history inferred using the Maximum Likelihood method as calculated using RAxML [S113] for cyt *b*. Rapid bootstrapping was used followed by searching for the best ML Tree. Bootstrapping was performed using a random seed, 100 repetitions, a general time reversible model of nucleotide substitution [S124] with the I model of rate heterogeneity [S125] and four discrete rate categories. Tree length is 66.78724. The six reversible substitution rates, four stationary state frequencies (pi), the shape of the gamma distribution (α) and the proportion of invariable sites (pinvar) can be found in Table S4. Posterior probabilities are shown as symbols on the branches (for values >75%).
(10.27 MB EPS)

Figure S11 Legend for Figures S1–S10. Phylogenetic trees based on the aligned sequences for cyt *b* and COI of 236 mammals (compromising 29 Orders, 89 Families, 174 genera and 217 species). Color ranges are the same for all trees and correspond to the Order of the species. Minimum Evolution, Maximum Parsimony and Neighbor-Joining phylogenetic trees were created in MEGA 4.0 [S109–110]. Maximum Likelihood phylogenetic trees were calculated in MrBayes [S111–112] and RAxML [S113]. All trees were exported and edited online using the Interactive Tree Of Life (iTOL) [S114] to define color ranges and export as image files.
(9.07 MB EPS)

Figure S12 The *R_s_* values for COI (A) and cyt *b* (B) over the entire genes represented by the blue bars. A value of 2 represents 100% conservation over all species at that base. Moving averages of homology are shown for blocks of 101 bp (red) 401 bp (purple) and 601 bp (yellow).
(8.24 MB EPS)

Figure S13 Receiver Operator Characteristic (ROC) curves for COI and cyt *b*. A reference line is given. The two genes cannot be differentiated based on their ROC curves. Area under the curves and statistical results can be found in Table S5. Calculated in SPSS 17.0.0.
(1.26 MB EPS)

Table S1 The list of complete mitochondrial sequences used for the cyt *b* and COI alignments. Accession number, common name, Order, Family, species and reference as given by the NCBI listing are shown. DS: Direct Submission.
(0.40 MB DOC)

Table S2 The list of human (*Homo sapiens*), domestic cattle (*Bos taurus*) and domestic dog (*Canis familiaris*) complete mitochondrial sequences used for the cyt *b* and COI alignments. Accession number, ethnicity/breed (if known) and reference as given by the NCBI listing are shown.
(0.27 MB DOC)

Table S3 The statistical results from the Maximum Likelihood phylogenetic trees calculated using MrBayes [S111–112] from 15,002 trees sampled. Tree length, six reversible substitution rates, four stationary state frequencies (pi), the shape of the gamma distribution (α) and the proportion of invariable sites (pinvar) are displayed.
(0.07 MB DOC)

Table S4 The statistical results from the Maximum Likelihood phylogenetic trees calculated using RAxML [S113] from 100 trees sampled. Tree length, six reversible substitution rates, four stationary state frequencies (pi), the shape of the gamma distribution (α) and the proportion of invariable sites (pinvar) are displayed.
(0.03 MB DOC)

Table S5 The statistical results from the ROC curve (Figure S3). Calculated in SPSS 17.0.0.
(0.03 MB DOC)
