# Supplementary material for: Reconstructing Mammalian Phylogenies: A Detailed Comparison of the Cytochrome b and Cytochrome Oxidase Subunit I Mitochondrial Genes
Source: PLoS One. 2010 Nov 30;5(11):e14156. doi: 10.1371/journal.pone.0014156 (PMC2994770; doi:10.1371/journal.pone.0014156)
Supplement: Text S1 — Supplementary references. (0.05 MB DOC) [file pone.0014156.s001.doc]

# Supplementary References

S1. Murata Y, Nikaido M, Sasaki T, Cao Y, Fukumoto Y, et al. (2003) Afrotherian phylogeny as inferred from complete mitochondrial genomes. Molecular Phylogenetics and Evolution 28: 253-260.

S2. Arnason U, Adegoke JA, Gullberg A, Harley EH, Janke A, et al. (2008) Mitogenomic relationships of placental mammals and molecular estimates of their divergences. Gene 421: 37-51.

S3. Mouchaty SK, Gullberg A, Janke A, Arnason U (2000) Phylogenetic position of the Tenrecs (Mammalia: Tenrecidae) of Madagascar based on analysis of the complete mitochondrial genome sequence of *Echinops telfairi*. Zoologica Scripta 29: 307-317.

S4. Mereu P, Di Suni MP, Manca L, Masala B (2008) Complete nucleotide mtDNA sequence of Barbary sheep (*Ammotragus lervia*). DNA Sequence - The Journal of Sequencing and Mapping 19: 241-245.

S5. Lowe T, Eddy S (1997) tRNAscan-SE: a program for improved detection of transfer RNA genes in genomic sequence. Nucleic Acids Research 25: 955-964.

S6. Pietro P, Maria F, GianFranco G, Giuseppe E (2003) The complete nucleotide sequence of goat (*Capra hircus*) mitochondrial genome. DNA Sequence - The Journal of Sequencing and Mapping 14: 199-203.

S7. Hiendleder S, Lewalski H, Wassmuth R, Janke A (1998) The complete mitochondrial DNA sequence of the domestic sheep ( *Ovis aries* ) and comparison with the other major Ovine haplotype. Journal of Molecular Evolution 47: 441-448.

S8. Hiendleder S (1998) A low rate of replacement substitutions in two major *Ovis aries* mitochondrial genomes. Animal Genetics 29: 116-122.

S9. Hiendleder S, Mainz K, Plante Y, Lewalski H (1998) Analysis of mitochondrial DNA indicates that domestic sheep are derived from two different ancestral maternal sources: no evidence for contributions from urial and argali sheep. Journal of Heredity 89: 113-120.

S10. Xu SQ, Yang YZ, Zhou J, Jing GE, Chen YT, et al. (2005) A mitochondrial genome sequence of the Tibetan antelope (*Pantholops hodgsonii*) Genomics Proteomics & Bioinformatics 3: 5-17.

S11. Cui P, Ji R, Ding F, Qi D, Gao H, et al. (2007) A complete mitochondrial genome sequence of the wild two-humped camel (*Camelus bactrianus ferus*): an evolutionary history of camelidae. BMC Genomics 8: 241.

S12. Ursing BM, Slack KE, Arnason U (2000) Subordinal artiodactyl relationships in the light of phylogenetic analysis of 12 mitochondrial protein-coding genes. Zoologica Scripta 29: 83-88.

S13. Wada K, Nishibori M, Yokohama M (2007) The complete nucleotide sequence of mitochondrial genome in the Japanese Sika deer (*Cervus nippon*), and a phylogenetic analysis between Cervidae and Bovidae. Small Ruminant Research 69: 46-54.

S14. Ursing BM, Arnason U (1998) Analyses of mitochondrial genomes strongly support a hippopotamus-whale clade. Proceedings of the Royal Society B: Biological Sciences 265: 2251-2255.

S15. Wu G-S, Yao Y-G, Qu K-X, Ding Z-L, Li H, et al. (2007) Population phylogenomic analysis of mitochondrial DNA in wild boars and domestic pigs revealed multiple domestication events in East Asia. Genome Biology 8: R245.

S16. Lin C-S, Sun Y-L, Liu C-Y, Yang P-C, Chang L-C, et al. (1999) Complete nucleotide sequence of pig (*Sus scrofa*) mitochondrial genome and dating evolutionary divergence within Artiodactyla. Gene 236: 107-114.

S17. Arnason U, Gullberg A, Janke A, Kullberg M (2007) Mitogenomic analyses of caniform relationships. Molecular Phylogenetics and Evolution 45: 863-874.

S18. Yonezawa T, Nikaido M, Kohno N, Fukumoto Y, Okada N, et al. (2007) Molecular phylogenetic study on the origin and evolution of Mustelidae. Gene 396: 1-12.

S19. Kim KS, Lee SE, Jeong HW, Ha JH (1998) The complete nucleotide sequence of the domestic dog (*Canis familiaris*) mitochondrial genome. Molecular Phylogenetics and Evolution 10: 210-220.

S20. Björnerfeldt S, Webster MT, Vilà C (2006) Relaxation of selective constraint on dog mitochondrial DNA following domestication. Genome Research 16: 990-994.

S21. Arnason U, Gullberg A, Janke A, Kullberg M, Lehman N, et al. (2006) Pinniped phylogeny and a new hypothesis for their origin and dispersal. Molecular Phylogenetics and Evolution 41: 345-354.

S22. Burger PA, Steinborn R, Walzer C, Petit T, Mueller M, et al. (2004) Analysis of the mitochondrial genome of cheetahs (*Acinonyx jubatus*) with neurodegenerative disease. Gene 338: 111-119.

S23. Lopez JV, Cevario S, O'Brien SJ (1996) Complete nucleotide sequences of the domestic cat (*Felis catus*) mitochondrial genome and a transposed mtDNA tandem repeat (Numt) in the nuclear genome. Genomics 33: 229-246.

S24. Wu XB, Zheng T, Jiang ZG, Wei L (2007) The mitochondrial genome structure of the clouded leopard (*Neofelis nebulosa*). Genome 50: 252-257.

S25. Arnason U, Adegoke JA, Bodin K, Born EW, Esa YB, et al. (2002) Mammalian mitogenomic relationships and the root of the eutherian tree. Proceedings of the National Academy of Sciences of the United States of America 99: 8151-8156.

S26. Lin Y-H, McLenachan PA, Gore AR, Phillips MJ, Ota R, et al. (2002) Four new mitochondrial genomes and the increased stability of evolutionary trees of mammals from improved taxon sampling. Molecular Biology and Evolution 19: 2060-2070.

S27. Árnason Ú, Gullberg A (1993) Comparison between the complete mtDNA sequences of the blue and the fin whale, two species that can hybridize in nature. Journal of Molecular Evolution 37: 312-322.

S28. Árnason Ú, Johnsson E (1992) The complete mitochondrial DNA sequence of the harbor seal, *Phoca vitulina*. Journal of Molecular Evolution 34: 493-505.

S29. Peng R, Zeng B, Meng X, Yue B, Zhang Z, et al. (2007) The complete mitochondrial genome and phylogenetic analysis of the giant panda (Ailuropoda melanoleuca). Gene 397: 76-83.

S30. Yu L, Li Y-W, Ryder O, Zhang Y-P (2007) Analysis of complete mitochondrial genome sequences increases phylogenetic resolution of bears (Ursidae), a mammalian family that experienced rapid speciation. BMC Evolutionary Biology 7: 198.

S31. Delisle I, Strobeck C (2002) Conserved primers for rapid sequencing of the complete mitochondrial genome from carnivores, applied to three species of bears. Molecular Biology and Evolution 19: 357-361.

S32. Hou W, Chen Y, Wu X, Hu J, Peng Z, et al. (2007) A complete mitochondrial genome sequence of Asian black bear Sichuan subspecies (*Ursus thibetanus mupinensis*). International Journal of Biological Sciences 3: 85-90.

S33. Hwang D-S, Ki J-S, Jeong D-H, Kim B-H, Lee B-K, et al. (2008) A comprehensive analysis of three Asiatic black bear mitochondrial genomes (subspecies *ussuricus*, *formosanus* and *mupinensis*), with emphasis on the complete mtDNA sequence of *Ursus thibetanus ussuricus* (Ursidae). Mitochondrial DNA 19: 418 - 429.

S34. Arnason U, Gullberg A, Janke A (2004) Mitogenomic analyses provide new insights into cetacean origin and evolution. Gene 333: 27-34.

S35. Sasaki T, Nikaido M, Hamilton H, Goto M, Kato H, et al. (2005) Mitochondrial phylogenetics and evolution of mysticete whales. Systematic Biology 54: 77-90.

S36. Sasaki T, Nikaido M, Wada S, Yamada TK, Cao Y, et al. (2006) Balaenoptera omurai is a newly discovered baleen whale that represents an ancient evolutionary lineage. Molecular Phylogenetics and Evolution 41: 40-52.

S37. Arnason U, Gullberg A, Widegren B (1993) Cetacean mitochondrial DNA control region: sequences of all extant baleen whales and two sperm whale species. Molecular Biology and Evolution 10: 960-970.

S38. Arnason U, Gullberg A, Widegren B (1991) The complete nucleotide sequence of the mitochondrial DNA of the fin whale, *Balaenoptera physalus*. Journal of Molecular Evolution 33: 556-568.

S39. Valverde JR, Marco R, Garesse R (1994) A conserved heptamer motif for ribosomal RNA transcription termination in animal mitochondria. Proceedings of the National Academy of Sciences of the United States of America 91: 5368-5371.

S40. Yan J, Zhou K, Yang G (2005) Molecular phylogenetics of 'river dolphins' and the baiji mitochondrial genome. Molecular Phylogenetics and Evolution 37: 743-750.

S41. Arnason U, Gullberg A, Gretarsdottir S, Ursing B, Janke A (2000) The mitochondrial genome of the sperm whale and a new molecular reference for estimating eutherian divergence dates. Journal of Molecular Evolution 50: 569-578.

S42. Pumo DE, Finamore PS, Franek WR, Phillips CJ, Tarzami S, et al. (1998) Complete mitochondrial genome of a neotropical fruit bat, *Artibeus jamaicensis*, and a new hypothesis of the relationships of bats to other Eutherian mammals. Journal of Molecular Evolution 47: 709-717.

S43. Nikaido M, Harada M, Cao Y, Hasegawa M, Okada N (2000) Monophyletic origin of the order Chiroptera and its phylogenetic position among Mammalia, as inferred from the complete sequence of the mitochondrial DNA of a Japanese megabat, the Ryukyu flying fox ( *Pteropus dasymallus* ). Journal of Molecular Evolution 51: 318-328.

S44. Lin Y-H, Penny D (2001) Implications for bat evolution from two new complete mitochondrial genomes. Molecular Biology and Evolution 18: 684-688.

S45. Nikaido M, Kawai K, Cao Y, Harada M, Tomita S, et al. (2001) Maximum likelihood analysis of the complete mitochondrial genomes of Eutherians and a reevaluation of the phylogeny of bats and insectivores. Journal of Molecular Evolution 53: 508-516.

S46. Arnason U, Gullberg A, Janke A (1997) Phylogenetic analyses of mitochondrial DNA suggest a sister group relationship between Xenarthra (Edentata) and Ferungulates. Molecular Biology and Evolution 14: 762-768.

S47. Phillips MJ, McLenachan PA, Down C, Gibb GC, Penny D (2006) Combined mitochondrial and nuclear DNA sequences resolve the interrelations of the major Australasian marsupial radiations. Systematic Biology 55: 122-137.

S48. Nilsson MA, Arnason U, Spencer PBS, Janke A (2004) Marsupial relationships and a timeline for marsupial radiation in South Gondwana. Gene 340: 189-196.

S49. Janke A, Feldmaier-Fuchs G, Thomas WK, von-Haeseler A, Paabo S (1994) The marsupial mitochondrial genome and the evolution of placental mammals. Genetics 137: 243-256.

S50. Nilsson MA, Gullberg A, Spotorno A, Arnason U, Janke A (2003) Radiation of extant marsupials after the K/T boundary: evidence from complete mitochondrial genomes. Journal of Molecular Evolution 57: S3-S12.

S51. Munemasa M, Nikaido M, Donnellan S, Austin CC, Okada N, et al. (2006) Phylogenetic analysis of Diprotodontian marsupials based on complete mitochondrial genomes. Genes & Genetic Systems 81: 181-191.

S52. Nilsson MA (2006) Phylogenetic relationships of the Banded Hare wallaby (*Lagostrophus fasciatus*) and a map of the kangaroo mitochondrial control region. Zoologica Scripta 35: 387-393.

S53. Janke A, Xu X, Arnason U (1997) The complete mitochondrial genome of the wallaroo (Macropus robustus) and the phylogenetic relationship among Monotremata, Marsupialia, and Eutheria. Proceedings of the National Academy of Sciences of the United States of America 94: 1276-1281.

S54. Phillips MJ, Lin Y-H, Harrison GL, Penny D (2001) Mitochondrial genomes of a bandicoot and a brushtail possum confirm the monophyly of australidelphian marsupials. Proceedings of the Royal Society B: Biological Sciences 268: 1533-1538.

S55. Janke A, Magnell O, Wieczorek G, Westerman M, Arnason U (2002) Phylogenetic analysis of 18S rRNA and the mitochondrial genomes of the wombat, *Vombatus ursinus*, and the spiny anteater, *Tachyglossus aculeatus*: increased support for the marsupionta hypothesis. Journal of Molecular Evolution 54: 71-80.

S56. Krettek A, Gullberg A, Arnason U (1995) Sequence analysis of the complete mitochondrial DNA molecule of the hedgehog, *Erinaceus europaeus*, and the phylogenetic position of the Lipotyphla. Journal of Molecular Evolution 41: 952-957.

S57. Nikaido M, Cao Y, Harada M, Okada N, Hasegawa M (2003) Mitochondrial phylogeny of hedgehogs and monophyly of Eulipotyphla. Molecular Phylogenetics and Evolution 28: 276-284.

S58. Gissi C, Gullberg A, Arnason U (1998) The complete mitochondrial DNA sequence of the rabbit, *Oryctolagus cuniculus*. Genomics 50: 161-169.

S59. Lin Y-H, Waddell PJ, Penny D (2002) Pika and vole mitochondrial genomes increase support for both rodent monophyly and glires. Gene 294: 119-129.

S60. Axel J, Neil JG, Gertaud F-F, Arndt von H, Svante P (1996) The mitochondrial genome of a Monotreme—The Platypus (*Ornithorhynchus anatinus*). Journal of Molecular Evolution 42: 153-159.

S61. Xu X, Janke A, Arnason U (1996) The complete mitochondrial DNA sequence of the greater Indian rhinoceros, *Rhinoceros unicornis*, and the Phylogenetic relationship among Carnivora, Perissodactyla, and Artiodactyla (+ Cetacea). Molecular Biology and Evolution 13: 1167-1173.

S62. Xu X, Gullberg A, Arnason U (1996) The complete mitochondrial DNA (mtDNA) of the donkey and mtDNA comparisons among four closely related mammalian species-pairs. Journal of Molecular Evolution 43: 438-446.

S63. Xiufeng X, Árnason Ú (1994) The complete mitochondrial DNA sequence of the horse, *Equus caballus*: extensive heteroplasmy of the control region. Gene 148: 357-362.

S64. Xu X, Arnason U (1997) The complete mitochondrial DNA sequence of the white rhinoceros, *Ceratotherium simum*, and comparison with the mtDNA sequence of the Indian rhinoceros, *Rhinoceros unicornis*. Molecular Phylogenetics and Evolution 7: 189-194.

S65. Arnason U, Gullberg A, Burguete AS, Janice A (2000) Molecular estimates of primate divergences and new hypotheses for primate dispersal and the origin of modern humans*. Hereditas 133: 217-228.

S66. Wertheim JO, Worobey M (2007) A challenge to the ancient origin of SIVagm based on African green monkey mitochondrial genomes. PLoS Pathogens 3: e95.

S67. Raaum RL, Sterner KN, Noviello CM, Stewart C-B, Disotell TR (2005) Catarrhine primate divergence dates estimated from complete mitochondrial genomes: concordance with fossil and nuclear DNA evidence. Journal of Human Evolution 48: 237-257.

S68. Sterner KN, Raaum RL, Zhang Y-P, Stewart C-B, Disotell TR (2006) Mitochondrial data support an odd-nosed colobine clade. Molecular Phylogenetics and Evolution 40: 1-7.

S69. Arnason U, Gullberg A, Janke A (1998) Molecular timing of primate divergences as estimated by two nonprimate calibration points. Journal of Molecular Evolution 47: 718-727.

S70. Horai S, Hayasaka K, Kondo R, Tsugane K, Takahata N (1995) Recent African origin of modern humans revealed by complete sequences of hominoid mitochondrial DNAs. Proceedings of the National Academy of Sciences of the United States of America 92: 532-536.

S71. Xu X, Arnason U (1996) A complete sequence of the mitochondrial genome of the western lowland gorilla. Molecular Biology and Evolution 13: 691-698.

S72. Anderson S, Bankier AT, Barrell BG, de Bruijn MHL, Coulson AR, et al. (1981) Sequence and organization of the human mitochondrial genome. Nature 290: 457-465.

S73. Andrews RM, Kubacka I, Chinnery PF, Lightowlers RN, Turnbull DM, et al. (1999) Reanalysis and revision of the Cambridge reference sequence for human mitochondrial DNA. Nature Genetics 23: 147-147.

S74. Ingman M, Kaessmann H, Paabo S, Gyllensten U (2000) Mitochondrial genome variation and the origin of modern humans. Nature 408: 708-713.

S75. Horai S, Satta Y, Hayasaka K, Kondo R, Inoue T, et al. (1992) Man's place in hominoidea revealed by mitochondrial DNA genealogy. Journal of Molecular Evolution 35: 32-43.

S76. Foran DR, Hixson JE, Brown WM (1988) Comparisons of ape and human sequences that regulate mitochondrial DNA transcription and D-loop DNA synthesis. Nucleic Acids Research 16: 5841-5861.

S77. Hixson J, Brown W (1986) A comparison of the small ribosomal RNA genes from the mitochondrial DNA of the great apes and humans: sequence, structure, evolution, and phylogenetic implications. Molecular Biology and Evolution 3: 1-18.

S78. Xu X, Arnason U (1996) The mitochondrial DNA molecule of sumatran orangutan and a molecular proposal for two (Bornean and Sumatran) species of orangutan. Journal of Molecular Evolution 43: 431-437.

S79. Arnason U, Gullberg A, Xu X (1996) A complete mitochondrial DNA molecule of the white-handed gibbon, *Hylobates lar*, and comparison among individual mitochondrial genes of all hominoid genera. Hereditas 124: 185-189.

S80. Matsui A, Rakotondraparany F, Hasegawa M, Horai S (2007) Determination of a complete lemur mitochondrial genome from feces. Mammal Study 32: 7-16.

S81. Schmitz J, Ohme M, Zischler H (2002) The complete mitochondrial sequence of *Tarsius bancanus*: evidence for an extensive nucleotide compositional plasticity of primate mitochondrial DNA. Molecular Biology and Evolution 19: 544-553.

S82. Rogaev EI, Moliaka YK, Malyarchuk BA, Kondrashov FA, Derenko MV, et al. (2006) Complete mitochondrial genome and phylogeny of pleistocene mammoth *Mammuthus primigenius*. PLoS Biology 4: e73.

S83. Hauf J, Waddell PJ, Chalwatzis N, Joger U, Zimmermann FK (2000) The complete mitochondrial genome sequence of the African elephant (*Loxodonta africana*), phylogenetic relationships of Proboscidea to other mammals and D-loop heteroplasmy. Zoology 102: 184-195.

S84. D'Erchia AM, Gissi C, Pesole G, Saccone C, Arnason U (1996) The guinea-pig is not a rodent. Nature 381: 597-600.

S85. Partridge MA, Davidson MM, Hei TK (2007) The complete nucleotide sequence of Chinese hamster (Cricetulus griseus) mitochondrial DNA. DNA Sequence - The Journal of Sequencing and Mapping 18: 341-346.

S86. Triant D, DeWoody J (2006) Accelerated molecular evolution in Microtus (Rodentia) as assessed via complete mitochondrial genome sequences. Genetica 128: 95-108.

S87. Reyes A, Pesole G, Saccone C (1998) Complete mitochondrial DNA sequence of the fat dormouse, *Glis glis*: further evidence of rodent paraphyly. Molecular Biology and Evolution 15: 499-505.

S88. Bayona-Bafaluy MP, Acin-Perez R, Mullikin JC, Park JS, Moreno-Loshuertos R, et al. (2003) Revisiting the mouse mitochondrial DNA sequence. Nucleic Acids Research 31: 5349-5355.

S89. Akimoto M, Niikura M, Ichikawa M, Yonekawa H, Nakada K, et al. (2005) Nuclear DNA but not mtDNA controls tumor phenotypes in mouse cells. Biochemical and Biophysical Research Communications 327: 1028-1035.

S90. Gregorova S, Divina P, Storchova R, Trachtulec Z, Fotopulosova V, et al. (2008) Mouse consomic strains: Exploiting genetic divergence between Mus m. musculus and Mus m. domesticus subspecies. Genome Research 18: 509-515.

S91. Gadaleta G, Pepe G, De Candia G, Quagliariello C, Sbisa E, et al. (1989) The complete nucleotide sequence of the *Rattus norvegicus* mitochondrial genome: Cryptic signals revealed by comparative analysis between vertebrates. Journal of Molecular Evolution 28: 497-516.

S92. Grosskopf R, Feldmann H (1981) Analysis of a DNA segment from rat liver mitochondria containing the genes for the cytochrome oxidase subunits I, II and III, ATPase subunit 6, and several tRNA genes. Current Genetics 4: 151-158.

S93. Grosskopf R, Feldmann H (1981) tRNA genes in rat liver mitochondrial DNA. Current Genetics 4: 191-196.

S94. Reyes A, Gissi C, Pesole G, Catzeflis FM, Saccone C (2000) Where do rodents fit? Evidence from the complete mitochondrial genome of *Sciurus vulgaris*. Molecular Biology and Evolution 17: 979-983.

S95. Mouchaty SK, Catzeflis F, Janke A, Arnason U (2001) Molecular evidence of an African phiomorpha-South American caviomorpha clade and support for hystricognathi based on the complete mitochondrial genome of the cane rat (*Thryonomys swinderianus*). Molecular Phylogenetics and Evolution 18: 127-135.

S96. Schmitz J, Ohme M, Zischler H (2000) The complete mitochondrial genome of *Tupaia belangeri* and the phylogenetic affiliation of Scandentia to other Eutherian orders. Molecular Biology and Evolution 17: 1334-1343.

S97. Fontanillas P, Dépraz A, Giorgi MS, Perrin N (2005) Nonshivering thermogenesis capacity associated to mitochondrial DNA haplotypes and gender in the greater white-toothed shrew, *Crocidura russula*. Molecular Ecology 14: 661-670.

S98. Cabria MT, Rubines J, Gómez-Moliner B, Zardoya R (2006) On the phylogenetic position of a rare Iberian endemic mammal, the Pyrenean desman (*Galemys pyrenaicus*). Gene 375: 1-13.

S99. Mouchaty SK, Gullberg A, Janke A, Arnason U (2000) The phylogenetic position of the Talpidae within Eutheria based on analysis of complete mitochondrial sequences. Molecular Biology and Evolution 17: 60-67.

S100. Arnason U, Gullberg A, Janke A (1999) The mitochondrial DNA molecule of the aardvark, *Orycteropus afer*, and the position of the Tubulidentata in the eutherian tree. Proceedings of the Royal Society B: Biological Sciences 266: 339-345.

S101. Behar DM, Villems R, Soodyall H, Blue-Smith J, Pereira L, et al. (2008) The dawn of human matrilineal diversity. American Journal of Human Genetics 82: 1130-1140.

S102. Quintana-Murci L, Quach H, Harmant C, Luca F, Massonnet B, et al. (2008) Maternal traces of deep common ancestry and asymmetric gene flow between Pygmy hunter gatherers and Bantu-speaking farmers. Proceedings of the National Academy of Sciences of the United States of America 105: 1596-1601.

S103. Behar DM, Metspalu E, Kivisild T, Rosset S, Tzur S, et al. (2008) Counting the founders: The matrilineal genetic ancestry of the Jewish diaspora. PLoS ONE 3: e2062.

S104. Coble MD, Just RS, O’Callaghan JE, Letmanyi IH, Peterson CT, et al. (2004) Single nucleotide polymorphisms over the entire mtDNA genome that increase the power of forensic testing in Caucasians. International Journal of Legal Medicine 118: 137-146.

S105. Just RS, Diegoli TM, Saunier JL, Irwin JA, Parsons TJ (2008) Complete mitochondrial genome sequences for 265 African American and U.S. "Hispanic" individuals. Forensic Science International: Genetics 2: e45-e48.

S106. Hiendleder S, Lewalski H, Janke A (2008) Complete mitochondrial genomes of Bos taurus and Bos indicus provide new insights into intra-species variation, taxonomy and domestication. Cytogenetic and Genome Research 120: 150-156.

S107. Achilli A, Olivieri A, Pellecchia M, Uboldi C, Colli L, et al. (2008) Mitochondrial genomes of extinct aurochs survive in domestic cattle. Current Biology 18: R157-R158.

S108. Anderson S, de Bruijn MHL, Coulson AR, Eperon IC, Sanger F, et al. (1982) Complete sequence of bovine mitochondrial DNA conserved features of the mammalian mitochondrial genome. Journal of Molecular Biology 156: 683-717.

S109. Kumar S, Nei M, Dudley J, Tamura K (2008) MEGA: A biologist-centric software for evolutionary analysis of DNA and protein sequences. Briefings in Bioinformatics 9: 299-306.

S110. Tamura K, Dudley J, Nei M, Kumar S (2007) MEGA4: Molecular Evolutionary Genetics Analysis (MEGA) software version 4.0. Molecular Biology and Evolution 24: 1596-1599.

S111. Huelsenbeck JP, Ronquist F (2001) MRBAYES: Bayesian inference of phylogenetic trees. Bioinformatics 17: 754-755.

S112. Ronquist F, Huelsenbeck JP (2003) MrBayes 3: Bayesian phylogenetic inference under mixed models. Bioinformatics 19: 1572-1574.

S113. Stamatakis A, Hoover P, Rougemont J (2008) A Rapid Bootstrap Algorithm for the RAxML Web Servers. Systematic Biology 57: 758-771.

S114. Letunic I, Bork P (2007) Interactive Tree Of Life (iTOL): an online tool for phylogenetic tree display and annotation. Bioinformatics 23: 127-128.

S115. Rzhetsky A, Nei M (1992) A simple method for estimating and testing Minimum-Evolution trees. Molecular Biology and Evolution 9: 945-967.

S116. Felsenstein J (1985) Confidence limits on phylogenies: An approach using the bootstrap. Evolution 39: 783-791.

S117. Kimura M (1980) A simple method for estimating evolutionary rates of base substitutions through comparative studies of nucleotide sequences. Journal of Molecular Evolution 16: 111-120.

S118. Nei M, Kumar S (2000) Molecular Evolution and Phylogenetics. New York: Oxford University Press. 333 p.

S119. Saitou N, Nei M (1987) The neighbor-joining method: a new method for reconstructing phylogenetic trees. Molecular Biology and Evolution 4: 406-425.

S120. Eck RV, Dayhoff MO (1966) Atlas of Protein Sequence and Structure. Silver Springs, Maryland: National Biomedical Research Foundation.

S121. Geyer CJ. Markov chain Monte Carlo maximum likelihood. In: Keramidas EM, editor. Proceedings of the 23rd Symposium on the Interface; 1991; Fairfax Station: Interface Foundation. pp. 156-163.

S122. Goldman N, Yang Z (1994) A codon-based model of nucleotide substitution for protein-coding DNA sequences. Molecular Biology and Evolution 11: 725-736.

S123. Muse SV, Gaut BS (1994) A likelihood approach for comparing synonymous and nonsynonymous nucleotide substitution rates, with application to the chloroplast genome. Molecular Biology and Evolution 11: 715-724.

S124. Tavare S (1986) Some probabilistic and statisical problems on the analysis of DNA sequences. Lect Math Life Sci 17: 57-86.

S125. Yang Z (1996) Among-site rate variation and its impact on phylogenetic analyses. Trends in Ecology & Evolution 11: 367-372.
