# Supplementary material for: Reconstructing Mammalian Phylogenies: A Detailed Comparison of the Cytochrome b and Cytochrome Oxidase Subunit I Mitochondrial Genes
Source: PLoS One. 2010 Nov 30;5(11):e14156. doi: 10.1371/journal.pone.0014156 (PMC2994770; doi:10.1371/journal.pone.0014156)
Supplement: Text S2 — Supplementary Figure 2 caption. (0.03 MB DOC) [file pone.0014156.s002.doc]

# Supplementary Figure 2 Caption

Figure 2: Split Orders are as follows:

Cytochrome *b* tree: Diprotodontia 1 (Long-nosed Potoroo, Banded Hare-wallaby, Wallaroo and Rufous Hare-wallaby); Diprotodontia 2 (New Guinea Feather-tailed Possum); Diprotodontia 3 (Common Ring-tailed Possum); Diprotodontia 4 (Honey Possum); Diprotodontia 5 (Sugar Glider and Striped Possum); Diprotodontia 6 (Stein’s Cuscus, Silver-gray Brushtail Possum, Common Wombat and Koala); Rodentia 1 (Lesser Egyptian Jerboa); Rodentia 2 (Ehrenberg’s Molerat, Chinese Hamster, Southern Vole, Taiwan Vole, Rat (Wistar), Rat (BN/SsNHsdMCW), Earth-colored Mouse, Eastern European House Mouse, Japanese Wild Mouse, House Mouse and Western European House Mouse); Rodentia 3 (Fat Dormouse and Eurasian Red Squirrel); Rodentia 4 (Greater Cane Rat and Guinea-pig); Afrosoricida 1 (Cape Golden Mole and Grant’s Golden Mole); Afrosoricida 2 (Lesser Hedgehog Tenrec); Primates 1 (Horsfields Tarsier); Primates 2 (White-fronted Capuchin, Green Monkey, Tantalus Monkey, Vervet Monkey, Guereza, Barbary Ape, Proboscis Monkey, Hamadryas Baboon, Mitred Leaf Monkey, Red Colobus, Douc Langur, Golden Snub-nosed Monkey, Hanuman Langur, Dusky Leaf Monkey, Western Gorilla, Western Lowland Gorilla, Human, Human 2, Chimpanzee, Bonobo, Bornean Orangutan, Sumatran Orangutan and Lar Gibbon); Primates 3 (Aye-aye, Slow Loris, Coquerel’s Sifaka, Mongoose Lemur and Ring-tailed Lemur); Artiodactyla 1 (Warthog and Domestic Pig), Artiodactyla 2 (Barbary Sheep, Domestic Cow, Domestic Yak, Zebu Cattle, Swamp Buffalo, Domestic Goat, Taiwan Serow, Domestic Sheep, Chiru, Formosan Sambar, Formosan Sika Deer, Hokkaido Sika Deer, Hondo Sika Deer, Red Deer, Yakushima Sika Deer, Tufted Deer, Black Muntjac, Chinese Muntjac, Formosan Muntjac, Indian Muntjac and Reindeer) and; Artiodactyla 3 (Alpaca, Hippopotamus, Arabian Camel, Wild Bactrian Camel and Bactrian Camel).

Cytochrome Oxidase I tree: Diprotodontia 1 (Honey Possum); Diprotodontia 2 (Stein’s Cuscus, Silver-gray Brushtail Possum, Long-nosed Potoroo, Banded Hare-wallaby, Wallaroo and Rufous Hare-wallaby); Diprotodontia 3 (Common Wombat and Koala); Diprotodontia 4 (Common Ring-tailed Possum, Sugar Glider and Striped Possum); Diprotodontia 5 (New Guinea Feather-tailed Possum); Rodentia 1 (Fat Dormouse); Rodentia 2 (Ehrenberg’s Molerat, Lesser Egyptian Jerboa, Chinese Hamster, Southern Vole, Taiwan Vole, Rat (Wistar), Rat (BN/SsNHsdMCW), Earth-colored Mouse, Eastern European House Mouse, Japanese Wild Mouse, House Mouse and Western European House Mouse); Rodentia 3 (Greater Cane Rat and Guinea-pig); Rodentia 4 (Eurasian Red Squirrel); Primates 1 (Horsfield’s tarsier); Primates 2 (White-Fronted Capuchin, Green Monkey, Tantalus Monkey, Vervet Monkey, Guereza, Barbary Ape, Proboscis Monkey, Hamadryas Baboon, Mitred Leaf Monkey, Red Colobus, Douc Langur, Golden Snub-nosed Monkey, Hanuman Langur, Dusky Leaf Monkey, Western Gorilla, Western Lowland Gorilla, Human, Human 2, Chimpanzee, Bonobo, Bornean Orangutan, Sumatran Orangutan and Lar Gibbon); Primates 3 (Slow Loris); Primates 4 (Aye-aye, Coquerel’s Sifaka, Mongoose Lemur and Ring-tailed Lemur); Lagomorphia 1 (Rabbit and European Hare); Lagomorphia 2 (Black-lipped Pika, American Pika and Collared Pika); Afrosoricida 1 (Cape Golden Mole and Grant’s Golden Mole); Afrosoricida 2 (Lesser Hedgehog Tenrec); Artiodactyla 1 (Barbary Sheep, Domestic Cow, Domestic Yak, Zebu Cattle, Swamp Buffalo, Domestic Goat, Taiwan Serow, Domestic Sheep, Chiru, Formosan Sambar, Formosan Sika Deer, Hokkaido Sika Deer, Hondo Sika Deer, Red Deer, Yakushima Sika Deer, Tufted Deer, Black Muntjac, Chinese Muntjac, Formosan Muntjac, Indian Muntjac and Reindeer); Artiodactyla 2 (Warthog and Domestic Pig); Artiodactyla 3 (Alpaca, Arabian Camel, Wild Bactrian Camel and Bactrian Camel) and; Artiodactyla 4 (Hippopotamus).
