# Supplementary material for: Reconstructing Mammalian Phylogenies: A Detailed Comparison of the Cytochrome b and Cytochrome Oxidase Subunit I Mitochondrial Genes
Source: PLoS One. 2010 Nov 30;5(11):e14156. doi: 10.1371/journal.pone.0014156 (PMC2994770; doi:10.1371/journal.pone.0014156)
Supplement: Table S1 — The list of complete mitochondrial sequences used for the cyt b and COI alignments. Accession number, common name, Order, Family, species and reference as given by the NCBI listing are shown. DS: Direct Submission. (0.40 MB DOC) [file pone.0014156.s016.doc]

| **NCBI** | **Common Name** | **Order** | **Family** | **Species** | **Reference** |
| --- | --- | --- | --- | --- | --- |
| NC_004920 | Cape Golden Mole | Afrosoricida | Chrysochloridae | *Chrysochloris asiatica* | [S1] |
| NC_010304 | Grant's Golden Mole | Afrosoricida | Chrysochloridae | *Eremitalpa granti* | [S2] |
| NC_002631 | Lesser Hedgehog Tenrec | Afrosoricida | Tenrecidae | *Echinops telfairi* | [S3] |
| NC_009510 | Barbary Sheep | Artiodactyla | Bovidae | *Ammotragus lervia* | [S4] |
| NC_006380 | Domestic Yak | Artiodactyla | Bovidae | *Bos grunniens* | DS |
| NC_005971 | Zebu Cattle | Artiodactyla | Bovidae | *Bos indicus* | DS |
| NC_006853 | Domestic Cattle | Artiodactyla | Bovidae | *Bos taurus* | [S5] |
| NC_006295 | Swamp Buffalo | Artiodactyla | Bovidae | *Bubalus bubalis* | DS |
| NC_005044 | Domestic Goat | Artiodactyla | Bovidae | *Capra hircus* | [S6] |
| NC_010640 | Taiwan Serow | Artiodactyla | Bovidae | *Naemorhedus swinhoei* | DS |
| NC_001941 | Domestic Sheep | Artiodactyla | Bovidae | *Ovis aries* | [S7-S9] |
| NC_007441 | Chiru | Artiodactyla | Bovidae | *Pantholops hodgsonii* | [S10] |
| NC_009628 | Bactrian Camel | Artiodactyla | Camelidae | *Camelus bactrianus* | [S11] |
| NC_009629 | Wild Bactrian Camel | Artiodactyla | Camelidae | *Camelus ferus* | [S11] |
| NC_009849 | Arabian Camel | Artiodactyla | Camelidae | *Camelus dromedarius* | DS |
| NC_002504 | Alpaca | Artiodactyla | Camelidae | *Vicugna pacos* | [S12] |
| NC_007704 | Red Deer | Artiodactyla | Cervidae | *Cervus elaphus* | DS |
| NC_006993 | Hondo Sika Deer | Artiodactyla | Cervidae | *Cervus nippon centralis* | DS |
| NC_008462 | Formosan Sika Deer | Artiodactyla | Cervidae | *Cervus nippon taiouanus* | DS |
| NC_007179 | Yakushima Sika Deer | Artiodactyla | Cervidae | *Cervus nippon yakushimae* | DS |
| NC_006973 | Hokkaido Sika Deer | Artiodactyla | Cervidae | *Cervus nippon yesoensis* | [S13] |
| NC_008749 | Tufted Deer | Artiodactyla | Cervidae | *Elaphodus cephalophus* | DS |
| NC_004577 | Black Muntjac | Artiodactyla | Cervidae | *Muntiacus crinifrons* | DS |
| NC_004563 | Indian Muntjac | Artiodactyla | Cervidae | *Muntiacus muntjak* | DS |
| NC_004069 | Chinese Muntjac | Artiodactyla | Cervidae | *Muntiacus reevesi* | DS |
| NC_008491 | Formosan Muntjac | Artiodactyla | Cervidae | *Muntiacus reevesi micrurus* | DS |
| NC_007703 | Reindeer | Artiodactyla | Cervidae | *Rangifer tarandus* | DS |
| NC_008414 | Formosan Sambar | Artiodactyla | Cervidae | *Rucervus unicolor swinhoei* | DS |
| NC_000889 | Hippopotamus | Artiodactyla | Hippopotamidae | *Hippopotamus amphibius* | [S14] |
| NC_008830 | Warthog | Artiodactyla | Suidae | *Phacochoerus africanus* | [S15] |
| NC_000845 | Domestic Pig | Artiodactyla | Suidae | *Sus domesticus* | [S16] |
| NC_011124 | Red Panda | Carnivora | Ailuridae | *Ailurus fulgens* | [S17] |
| NC_009691 | Red Panda | Carnivora | Ailuridae | *Ailurus fulgens styani* | [S18] |
| NC_002008 | Domestic Dog | Carnivora | Canidae | *Canis familiaris* | [S19] |
| NC_008093 | Coyote | Carnivora | Canidae | *Canis latrans* | [S20] |
| NC_008092 | Gray Wolf | Carnivora | Canidae | *Canis lupus* | [S20] |
| NC_010340 | Mongolian Wolf | Carnivora | Canidae | *Canis lupus chanco* | DS |
| NC_011218 | Tibetan Wolf | Carnivora | Canidae | *Canis lupus laniger* | DS |
| NC_009686 | Eurasian Wolf | Carnivora | Canidae | *Canis lupus lupus* | [S17] |
| NC_008434 | Red Fox | Carnivora | Canidae | *Vulpes vulpes* | [S21] |
| NC_005212 | Cheetah | Carnivora | Felidae | *Acinonyx jubatus* | [S22] |
| NC_001700 | Domestic Cat | Carnivora | Felidae | *Felis catus* | [S23] |
| NC_008450 | Clouded Leopard | Carnivora | Felidae | *Neofelis nebulosa* | [S24] |
| NC_010641 | Leopard | Carnivora | Felidae | *Panthera pardus* | DS |
| NC_010642 | Tiger | Carnivora | Felidae | *Panthera tigris* | DS |
| NC_010638 | Snow Leopard | Carnivora | Felidae | *Panthera uncia* | DS |
| NC_006835 | Small Indian Mongoose | Carnivora | Herpestidae | *Herpestes auropunctatus* | DS |
| NC_010497 | Eastern Spotted Skunk | Carnivora | Mephitidae | *Spilogale putorius* | [S17] |
| NC_009692 | Sea Otter | Carnivora | Mustelidae | *Enhydra lutris* | [S18] |
| NC_009685 | Wolverine | Carnivora | Mustelidae | *Gulo gulo* | [S17] |
| NC_011358 | Eurasian Otter | Carnivora | Mustelidae | *Lutra lutra* | DS |
| NC_009678 | Japanese Marten | Carnivora | Mustelidae | *Martes melampus* | [S18] |
| NC_009677 | Japanese Badger | Carnivora | Mustelidae | *Meles anakuma* | [S18] |
| NC_011125 | Eurasian Badger | Carnivora | Mustelidae | *Meles meles* | [S17] |
| NC_004029 | Atlantic Walrus | Carnivora | Odobenidae | *Odobenus rosmarus rosmarus* | [S25] |
| NC_004023 | New Zealand Fur Seal | Carnivora | Otariidae | *Arctocephalus forsteri* | [S26] |
| NC_008417 | Cape Fur Seal | Carnivora | Otariidae | *Arctocephalus pusillus* | [S21] |
| NC_008420 | Guadalupe Fur Seal | Carnivora | Otariidae | *Arctocephalus townsendi* | [S21] |
| NC_008415 | Northern Fur Seal | Carnivora | Otariidae | *Callorhinus ursinus* | [S21] |
| NC_004030 | Steller’s Sealion | Carnivora | Otariidae | *Eumetopias jubatus* | [S25] |
| NC_008419 | Australian Sealion | Carnivora | Otariidae | *Neophoca cinerea* | [S21] |
| NC_008418 | Hooker's Sealion | Carnivora | Otariidae | *Phocarctos hookeri* | [S21] |
| NC_008416 | California Sealion | Carnivora | Otariidae | *Zalophus californianus* | [S21] |
| NC_008427 | Hooded Seal | Carnivora | Phocidae | *Cystophora cristata* | [S21] |
| NC_008426 | Bearded Seal | Carnivora | Phocidae | *Erignathus barbatus* | [S21] |
| NC_001602 | Gray Seal | Carnivora | Phocidae | *Halichoerus grypus* | [S27] |
| NC_008428 | Ribbon Seal | Carnivora | Phocidae | *Histriophoca fasciata* | [S21] |
| NC_008425 | Leopard Seal | Carnivora | Phocidae | *Hydrurga leptonyx* | [S21] |
| NC_008424 | Weddell’s Seal | Carnivora | Phocidae | *Leptonychotes weddellii* | [S21] |
| NC_008423 | Crabeater Seal | Carnivora | Phocidae | *Lobodon carcinophaga* | [S21] |
| NC_008422 | Southern Elephant Seal | Carnivora | Phocidae | *Mirounga leonina* | [S21] |
| NC_008421 | Hawaiian Monk Seal | Carnivora | Phocidae | *Monachus schauinslandi* | [S21] |
| NC_008429 | Harp Seal | Carnivora | Phocidae | *Pagophilus groenlandicus* | [S21] |
| NC_008430 | Spotted Seal | Carnivora | Phocidae | *Phoca largha* | [S21] |
| NC_001325 | Harbor Seal | Carnivora | Phocidae | *Phoca vitulina* | [S28] |
| NC_008431 | Caspian Seal | Carnivora | Phocidae | *Pusa caspica* | [S21] |
| NC_008433 | Ringed Seal | Carnivora | Phocidae | *Pusa hispida* | [S21] |
| NC_008432 | Baikal Seal | Carnivora | Phocidae | *Pusa sibirica* | [S21] |
| NC_009126 | Raccoon | Carnivora | Procyonidae | *Procyon lotor* | DS |
| NC_009492 | Giant Panda | Carnivora | Ursidae | *Ailuropoda melanoleuca* | [S29] |
| NC_009968 | Malayan Sun Bear | Carnivora | Ursidae | *Helarctos malayanus* | [S30] |
| NC_009970 | Sloth Bear | Carnivora | Ursidae | *Melursus ursinus* | [S30] |
| NC_009969 | Spectacled Bear | Carnivora | Ursidae | *Tremarctos ornatus* | [S30] |
| NC_003426 | American Black Bear | Carnivora | Ursidae | *Ursus americanus* | [S31] |
| NC_003427 | Brown Bear | Carnivora | Ursidae | *Ursus arctos* | [S31] |
| NC_003428 | Polar Bear | Carnivora | Ursidae | *Ursus maritimus* | [S31] |
| NC_009971 | Asian Black Bear | Carnivora | Ursidae | *Ursus thibetanus* | [S30] |
| NC_009331 | Formosan Black Bear | Carnivora | Ursidae | *Ursus thibetanus formosanus* | DS |
| NC_008753 | Sichuan Black Bear | Carnivora | Ursidae | *Ursus thibetanus mupinensis* | [S32] |
| NC_011118 | Himalayan Black Bear | Carnivora | Ursidae | *Ursus thibetanus thibetanus* | DS |
| NC_011517 | Manchurian Black Bear | Carnivora | Ursidae | *Ursus thibetanus ussuricus* | [S33] |
| NC_005268 | Bowhead Whale | Cetacea | Balaenidae | *Balaena mysticetus* | [S34] |
| NC_006930 | Southern Right Whale | Cetacea | Balaenidae | *Eubalaena australis* | [S35] |
| NC_006931 | North Pacific Right Whale | Cetacea | Balaenidae | *Eubalaena japonica* | [S35] |
| NC_005271 | Minke Whale | Cetacea | Balaenopteridae | *Balaenoptera acutorostrata* | [S34] |
| NC_006926 | Antarctic Minke Whale | Cetacea | Balaenopteridae | *Balaenoptera bonaerensis* | [S35] |
| NC_006929 | Sei Whale | Cetacea | Balaenopteridae | *Balaenoptera borealis* | [S35] |
| NC_006928 | Bryde's Whale | Cetacea | Balaenopteridae | *Balaenoptera brydei* | DS |
| NC_007938 | Pygmy Bryde's Whale | Cetacea | Balaenopteridae | *Balaenoptera edeni* | [S36] |
| NC_001601 | Blue Whale | Cetacea | Balaenopteridae | *Balaenoptera musculus* | [S27, S37] |
| NC_007937 | Omura's Whale | Cetacea | Balaenopteridae | *Balaenoptera omurai* | [S36] |
| NC_001321 | Fin Whale | Cetacea | Balaenopteridae | *Balaenoptera physalus* | [S38-S39] |
| NC_006927 | Humpback Whale | Cetacea | Balaenopteridae | *Megaptera novaeangliae* | [S35] |
| NC_005278 | White-beaked Dolphin | Cetacea | Delphinidae | *Lagenorhynchus albirostris* | [S34] |
| NC_005270 | Gray Whale | Cetacea | Eschrichtiidae | *Eschrichtius robustus* | [S34] |
| NC_005276 | Boutu | Cetacea | Iniidae | *Inia geoffrensis* | [S34] |
| NC_007629 | Yangtze River Dolphin | Cetacea | Iniidae | *Lipotes vexillifer* | [S40] |
| NC_005277 | Franciscana | Cetacea | Iniidae | *Pontoporia blainvillei* | [S34] |
| NC_005279 | Narwhal | Cetacea | Monodontidae | *Monodon monoceros* | [S34] |
| NC_005269 | Pygmy Right Whale | Cetacea | Neobalaenidae | *Caperea marginata* | [S34] |
| NC_005280 | Harbor Porpoise | Cetacea | Phocoenidae | *Phocoena phocoena* | [S34] |
| NC_005272 | Pygmy Sperm Whale | Cetacea | Physeteridae | *Kogia breviceps* | [S34] |
| NC_002503 | Sperm Whale | Cetacea | Physeteridae | *Physeter catodon* | [S41] |
| NC_005275 | Indus River Dolphin | Cetacea | Platanistidae | *Platanista minor* | [S34] |
| NC_005274 | Baird's Beaked Whale | Cetacea | Ziphiidae | *Berardius bairdii* | [S34] |
| NC_005273 | Northern Bottle-nosed Whale | Cetacea | Ziphiidae | *Hyperoodon ampullatus* | [S34] |
| NC_006925 | New Zealand Lesser Short -tailed Bat | Chiroptera | Mystacinidae | *Mystacina tuberculata* | DS |
| NC_002009 | Jamaican Fruit Bat | Chiroptera | Phyllostomidae | *Artibeus jamaicensis* | [S42] |
| NC_002612 | Ryukyu Flying Fox | Chiroptera | Pteropodidae | *Pteropus dasymallus* | [S43] |
| NC_002619 | Little Red Flying Fox | Chiroptera | Pteropodidae | *Pteropus scapulatus* | [S44] |
| NC_007393 | Egyptian Fruit Bat | Chiroptera | Pteropodidae | *Rousettus aegyptiacus* | DS |
| NC_011304 | Formosan Woolly Horseshoe Bat | Chiroptera | Rhinolophidae | *Rhinolophus formosae* | DS |
| NC_005433 | Formosan Lesser Horseshoe Bat | Chiroptera | Rhinolophidae | *Rhinolophus monoceros* | [S26] |
| NC_005434 | Okinawa Least Horseshoe Bat | Chiroptera | Rhinolophidae | *Rhinolophus pumilus* | [S45] |
| NC_002626 | New Zealand Long-tailed Bat | Chiroptera | Vespertilionidae | *Chalinolobus tuberculatus* | [S44] |
| NC_005436 | Japanese House Bat | Chiroptera | Vespertilionidae | *Pipistrellus abramus* | [S45] |
| NC_001821 | Nine-banded Armadillo | Cingulata | Dasypodidae | *Dasypus novemcinctus* | [S46] |
| NC_007630 | Northern Quoll | Dasyuromorphia | Dasyuridae | *Dasyurus hallucatus* | [S47] |
| NC_006523 | Brush-tailed Phascogale | Dasyuromorphia | Dasyuridae | *Phascogale tapoatafa* | [S48] |
| NC_007631 | Fat-tailed Dunnart | Dasyuromorphia | Dasyuridae | *Sminthopsis crassicaudata* | [S47] |
| NC_006517 | Julia Creek Dunnart | Dasyuromorphia | Dasyuridae | *Sminthopsis douglasi* | [S48] |
| NC_004031 | Sunda Flying Lemur | Dermoptera | Cynocephalidae | *Galeopterus variegatus* | [S25] |
| NC_001610 | North American Opossum | Didelphimorphia | Didelphidae | *Didelphis virginiana* | [S49] |
| NC_006516 | Brown Four-eyed Opossum | Didelphimorphia | Didelphidae | *Metachirus nudicaudatus* | [S48] |
| NC_006299 | Gray Short-tailed Opossum | Didelphimorphia | Didelphidae | *Monodelphis domestica* | [S50] |
| NC_005825 | Elegant Fat-tailed Opossum | Didelphimorphia | Didelphidae | *Thylamys elegans* | [S50] |
| NC_008145 | New Guinean Feather-tailed Possum | Diprotodontia | Acrobatidae | *Distoechurus pennatus* | [S51] |
| NC_008136 | Rufous Hare-wallaby | Diprotodontia | Macropodidae | *Lagorchestes hirsutus* | [S51] |
| NC_008447 | Banded Hare-wallaby | Diprotodontia | Macropodidae | *Lagostrophus fasciatus* | [S52] |
| NC_001794 | Wallaroo | Diprotodontia | Macropodidae | *Macropus robustus* | [S53] |
| NC_008134 | Striped Possum | Diprotodontia | Petauridae | *Dactylopsila trivirgata* | [S51] |
| NC_008135 | Sugar Glider | Diprotodontia | Petauridae | *Petaurus breviceps* | [S51] |
| NC_008137 | Stein's Cuscus | Diprotodontia | Phalangeridae | *Phalanger interpositus* | DS |
| NC_003039 | Brush-tailed Possum | Diprotodontia | Phalangeridae | *Trichosurus vulpecula* | [S54] |
| NC_008133 | Koala | Diprotodontia | Phascolarctidae | *Phascolarctos cinereus* | [S51] |
| NC_006524 | Long-nosed Potoroo | Diprotodontia | Potoroidae | *Potorous tridactylus* | [S48] |
| NC_006519 | Common Ring-tailed Possum | Diprotodontia | Pseudocheiridae | *Pseudocheirus peregrinus* | [S48] |
| NC_006518 | Honey Possum | Diprotodontia | Tarsipedidae | *Tarsipes rostratus* | [S48] |
| NC_003322 | Common Wombat | Diprotodontia | Vombatidae | *Vombatus ursinus* | [S55] |
| NC_002808 | Moonrat | Erinaceomorpha | Erinaceidae | *Echinosorex gymnura* | DS |
| NC_002080 | Western European Hedgehog | Erinaceomorpha | Erinaceidae | *Erinaceus europaeus* | [S56] |
| NC_005033 | Long-eared Hedgehog | Erinaceomorpha | Erinaceidae | *Hemiechinus auritus* | [S57] |
| NC_010298 | Short-tailed Gymnure | Erinaceomorpha | Erinaceidae | *Hylomys suillus* | [S2] |
| NC_010301 | Western Tree Hyrax | Hyracoidea | Procaviidae | *Dendrohyrax dorsalis* | [S2] |
| NC_004919 | Cape Rock Hyrax | Hyracoidea | Procaviidae | *Procavia capensis* | [S1] |
| NC_004028 | European Hare | Lagomorpha | Leporidae | *Lepus europaeus* | [S25] |
| NC_001913 | Rabbit | Lagomorpha | Leporidae | *Oryctolagus cuniculus* | [S58] |
| NC_003033 | Collared Pika | Lagomorpha | Ochotonidae | *Ochotona collaris* | [S59] |
| NC_011029 | Black-lipped Pika | Lagomorpha | Ochotonidae | *Ochotona curzoniae* | DS |
| NC_005358 | American Pika | Lagomorpha | Ochotonidae | *Ochotona princeps* | DS |
| NC_004026 | Short-eared Elephant Shrew | Macroscelidea | Macroscelididae | *Macroscelideus proboscideus* | [S25] |
| NC_005826 | Monitos Del Monte | Microbiotheria | Microbiotheriidae | *Dromiciops gliroides* | [S50] |
| NC_000891 | Platypus | Monotremata | Ornithorhynchidae | *Ornithorhynchus anatinus* | [S60] |
| NC_003321 | Australian Echidna | Monotremata | Tachyglossidae | *Tachyglossus aculeatus* | [S55] |
| NC_006364 | Long-beaked Echidna | Monotremata | Tachyglossidae | *Zaglossus bruijni* | [S48] |
| NC_006522 | Marsupial Mole | Notoryctemorphia | Notoryctidae | *Notoryctes typhlops* | [S48] |
| NC_005828 | Silky Shrew Opossum | Paucituberculata | Caenolestidae | *Caenolestes fuliginosus* | [S50] |
| NC_005829 | Chilean Shrew Opossum | Paucituberculata | Caenolestidae | *Rhyncholestes raphanurus* | [S50] |
| NC_002746 | Northern Brown Bandicoot | Peramelemorphia | Peramelidae | *Isoodon macrourus* | [S54] |
| NC_006520 | Bilby | Peramelemorphia | Thylacomyidae | *Macrotis lagotis* | [S48] |
| NC_001788 | Domestic Donkey | Perissodactyla | Equidae | *Equus asinus* | [S61-S62] |
| NC_001640 | Domestic Horse | Perissodactyla | Equidae | *Equus caballus* | [S63] |
| NC_001808 | White Rhinoceros | Perissodactyla | Rhinocerotidae | *Ceratotherium simum* | [S64] |
| NC_001779 | Indian Rhinoceros | Perissodactyla | Rhinocerotidae | *Rhinoceros unicornis* | [S61] |
| NC_004027 | Long-tailed Pangolin | Pholidota | Manidae | *Manis tetradactyla* | [S25] |
| NC_006923 | Three-toed Sloth | Pilosa | Bradypodidae | *Bradypus tridactylus* | DS |
| NC_006924 | Southern Two-toed Sloth | Pilosa | Megalonychidae | *Choloepus didactylus* | DS |
| NC_004032 | Southern Tamandua | Pilosa | Myrmecophagidae | *Tamandua tetradactyla* | [S25] |
| NC_002763 | White-fronted Capuchin | Primates | Cebidae | *Cebus albifrons* | [S65] |
| NC_009747 | Vervet Monkey | Primates | Cercopithecidae | *Chlorocebus pygerythrus* | [S66] |
| NC_008066 | Green Monkey | Primates | Cercopithecidae | *Chlorocebus sabaeus* | DS |
| NC_009748 | Tantalus Monkey | Primates | Cercopithecidae | *Chlorocebus tantalus* | [S66] |
| NC_006901 | Guereza | Primates | Cercopithecidae | *Colobus guereza* | [S67] |
| NC_002764 | Barbary Macaque | Primates | Cercopithecidae | *Macaca sylvanus* | [S65] |
| NC_008216 | Proboscis Monkey | Primates | Cercopithecidae | *Nasalis larvatus* | [S68] |
| NC_001992 | Hamadryas Baboon | Primates | Cercopithecidae | *Papio hamadryas* | [S69] |
| NC_008219 | Red Colobus | Primates | Cercopithecidae | *Piliocolobus badius* | [S68] |
| NC_008217 | Mitred Leaf Monkey | Primates | Cercopithecidae | *Presbytis melalophos* | [S68] |
| NC_008220 | Douc Langur | Primates | Cercopithecidae | *Pygathrix nemaeus* | [S68] |
| NC_008218 | Golden Snub-nosed Monkey | Primates | Cercopithecidae | *Rhinopithecus roxellana* | [S68] |
| NC_008215 | Hanuman Langur | Primates | Cercopithecidae | *Semnopithecus entellus* | [S68] |
| NC_006900 | Dusky Leaf Monkey | Primates | Cercopithecidae | *Trachypithecus obscurus* | [S67] |
| NC_010299 | Aye-aye | Primates | Daubentoniidae | *Daubentonia madagascariensis* | [S2] |
| NC_001645 | Western Gorilla | Primates | Hominidae | *Gorilla gorilla* | [S70] |
| NC_011120 | Western Lowland Gorilla | Primates | Hominidae | *Gorilla gorilla gorilla* | [S71] |
| AC_000021 | Human | Primates | Hominidae | *Homo sapiens* | [S72-S73] |
| NC_001807 | Human | Primates | Hominidae | *Homo sapiens* | [S74] |
| NC_001644 | Bonobo | Primates | Hominidae | *Pan paniscus* | [S70, S75-S77] |
| NC_001643 | Chimpanzee | Primates | Hominidae | *Pan troglodytes* | [S70, S75-S77] |
| NC_002083 | Sumatran Orangutan | Primates | Hominidae | *Pongo abelii* | [S78] |
| NC_001646 | Bornean Orangutan | Primates | Hominidae | *Pongo pygmaeus* | [S70, S75] |
| NC_002082 | Lar Gibbon | Primates | Hylobatidae | *Hylobates lar* | [S79] |
| NC_011053 | Coquerel's Sifaka | Primates | Indriidae | *Propithecus coquereli* | [S80] |
| NC_010300 | Mongoose Lemur | Primates | Lemuridae | *Eulemur mongoz* | [S2] |
| NC_004025 | Ring-tailed Lemur | Primates | Lemuridae | *Lemur catta* | [S25] |
| NC_002765 | Slow Loris | Primates | Lorisidae | *Nycticebus coucang* | [S65] |
| NC_002811 | Horsfield's Tarsier | Primates | Tarsiidae | *Tarsius bancanus* | [S81] |
| NC_005129 | Asian Elephant | Proboscidea | Elephantidae | *Elephas maximus* | [S82] |
| NC_000934 | African Savanna Elephant | Proboscidea | Elephantidae | *Loxodonta africana* | [S83] |
| NC_000884 | Guinea-pig | Rodentia | Caviidae | *Cavia porcellus* | [S84] |
| NC_007936 | Chinese Hamster | Rodentia | Cricetidae | *Cricetulus griseus* | [S85] |
| NC_003041 | Taiwan Vole | Rodentia | Cricetidae | *Microtus kikuchii* | [S59] |
| NC_008064 | Southern Vole | Rodentia | Cricetidae | *Microtus rossiaemeridionalis* | [S86] |
| NC_005314 | Lesser Egyptian Jerboa | Rodentia | Dipodidae | *Jaculus jaculus* | DS |
| NC_001892 | Fat Dormouse | Rodentia | Gliridae | *Glis glis* | [S87] |
| NC_005089 | House Mouse | Rodentia | Muridae | *Mus musculus* | [S5, S88] |
| NC_006914 | Western European House Mouse | Rodentia | Muridae | *Mus musculus domesticus* | DS |
| NC_006915 | Japanese Wild Mouse | Rodentia | Muridae | *Mus musculus molossinus* | [S89] |
| NC_010339 | Eastern European House Mouse | Rodentia | Muridae | *Mus musculus musculus* | [S90] |
| NC_010650 | Earth-colored Mouse | Rodentia | Muridae | *Mus terricolor* | DS |
| NC_001665 | Rat Strain BN/Ssnhsdmcw | Rodentia | Muridae | *Rattus norvegicus* | DS |
| AC_000022 | Rat Strain Wistar | Rodentia | Muridae | *Rattus norvegicus* | [S91-S93] |
| NC_002369 | Eurasian Red Squirrel | Rodentia | Sciuridae | *Sciurus vulgaris* | [S94] |
| NC_005315 | Ehrenberg's Molerat | Rodentia | Spalacidae | *Spalax ehrenbergi* | DS |
| NC_002658 | Greater Cane Rat | Rodentia | Thryonomyidae | *Thryonomys swinderianus* | [S95] |
| NC_002521 | Northern Tree Shrew | Scandentia | Tupaiidae | *Tupaia belangeri* | [S96] |
| NC_003314 | Dugong | Sirenia | Dugongidae | *Dugong dugon* | [S25] |
| NC_010302 | Caribbean Manatee | Sirenia | Trichechidae | *Trichechus manatus* | [S2] |
| NC_006893 | White-toothed Shrew | Soricomorpha | Soricidae | *Crocidura russula* | [S97] |
| NC_003040 | Taiwan Brown-toothed Shrew | Soricomorpha | Soricidae | *Episoriculus fumidus* | DS |
| NC_005435 | Long-clawed Shrew | Soricomorpha | Soricidae | *Sorex unguiculatus* | [S45] |
| NC_008156 | Pyrenean Desman | Soricomorpha | Talpidae | *Galemys pyrenaicus* | [S98] |
| NC_005035 | Japanese Mole | Soricomorpha | Talpidae | *Mogera wogura* | [S57] |
| NC_002391 | European Mole | Soricomorpha | Talpidae | *Talpa europaea* | [S99] |
| NC_005034 | Japanese Shrew Mole | Soricomorpha | Talpidae | *Urotrichus talpoides* | [S57] |
| NC_002078 | Aardvark | Tubulidentata | Orycteropodidae | *Orycteropus afer* | [S100] |
