# Supplementary material for: Reconstructing Mammalian Phylogenies: A Detailed Comparison of the Cytochrome b and Cytochrome Oxidase Subunit I Mitochondrial Genes
Source: PLoS One. 2010 Nov 30;5(11):e14156. doi: 10.1371/journal.pone.0014156 (PMC2994770; doi:10.1371/journal.pone.0014156)
Supplement: Table S2 — The list of human (Homo sapiens), domestic cattle (Bos taurus) and domestic dog (Canis familiaris) complete mitochondrial sequences used for the cyt b and COI alignments. Accession number, ethnicity/breed (if known) and reference as given by the NCBI listing are shown. (0.27 MB DOC) [file pone.0014156.s017.doc]

| **NCBI** | **Ethnicity/Breed** | | **Reference** |
| --- | --- | --- | --- |
| ***Homo sapiens*** | | | |
| AC_000021 | | rRCS | [S72-S73] |
| EU092658 | | Bedouin | [S101] |
| EU092659 | | Druze | [S101] |
| EU092660 | | Yemenite Jew | [S101] |
| EU092661 | | Ethiopian Jew | [S101] |
| EU092662 | | Ethiopian Jew | [S101] |
| EU092663-EU092665 | | Bedouin | [S101] |
| EU092666 | | Ethiopian Jew | [S101] |
| EU092667 | | Moroccan Jew | [S101] |
| EU092668-EU092670 | | Ethiopian Jew | [S101] |
| EU092671 | | Moroccan Jew | [S101] |
| EU092672 | | Bedouin | [S101] |
| EU092673 | | Ethiopian Jew | [S101] |
| EU092674 | | Ethiopian Jew | [S101] |
| EU092675-EU092677 | | Palestinian | [S101] |
| EU092678 | | Yemenite Jew | [S101] |
| EU092679-EU092681 | | Palestinian | [S101] |
| EU092682 | | Libyan Jew | [S101] |
| EU092683-EU092685 | | Palestinian | [S101] |
| EU092686 | | Yemenite Jew | [S101] |
| EU092687 | | Romanian Jew | [S101] |
| EU092688 | | Shangaan | [S101] |
| EU092689 | | Shangaan | [S101] |
| EU092690 | | Chopi | [S101] |
| EU092691 | | Tswa | [S101] |
| EU092692 | | Ronga | [S101] |
| EU092693 | | Ronga | [S101] |
| EU092694 | | Nyungwe | [S101] |
| EU092695 | | Ronga | [S101] |
| EU092696 | | Ronga | [S101] |
| EU092697 | | Mozambique | [S101] |
| EU092698 | | Ronga | [S101] |
| EU092699 | | Ronga | [S101] |
| EU092700 | | Mozambique | [S101] |
| EU092701 | | Shangaan | [S101] |
| EU092702 | | Shangaan | [S101] |
| EU092703 | | Mozambique | [S101] |
| EU092704 | | Chopi | [S101] |
| EU092705 | | Mozambique | [S101] |
| EU092706 | | Shangaan | [S101] |
| EU092707 | | Mozambique | [S101] |
| EU092708 | | Ronga | [S101] |
| EU092709 | | Ndau | [S101] |
| EU092710 | | Dutch | [S101] |
| EU092711-EU092713 | | Portuguese | [S101] |
| EU092714 | | Beafada | [S101] |
| EU092715 | | Fula | [S101] |
| EU092716 | | FulaForro | [S101] |
| EU092717 | | Fula | [S101] |
| EU092718 | | Papel | [S101] |
| EU092719 | | FutaFula | [S101] |
| EU092720 | | FulaForro | [S101] |
| EU092721 | | Mandinga | [S101] |
| EU092722 | | Manjaco | [S101] |
| EU092723 | | Fula | [S101] |
| EU092724 | | Mandinga | [S101] |
| EU092725 | | Beafada | [S101] |
| EU092726 | | Cassanga | [S101] |
| EU092727 | | Bijago | [S101] |
| EU092728 | | BalMane | [S101] |
| EU092729 | | Fula | [S101] |
| EU092730 | | Felupe | [S101] |
| EU092731 | | Beafada | [S101] |
| EU092732 | | Bijago | [S101] |
| EU092733 | | Balanta | [S101] |
| EU092734 | | Mandinga | [S101] |
| EU092735 | | Balanta | [S101] |
| EU092736 | | Bijago | [S101] |
| EU092737-EU092810 | | No information | [S101] |
| EU092811 | | Arab | [S101] |
| EU092812 | | Arab | [S101] |
| EU092813 | | Arab/Berber | [S101] |
| EU092814 | | Arab | [S101] |
| EU092815 | | Berber | [S101] |
| EU092816-EU092818 | | Arab | [S101] |
| EU092819 | | Kabyle | [S101] |
| EU092820-EU092830 | | No information | [S101] |
| EU092831-EU092839 | | San | [S101] |
| EU092840-EU092848 | | Khoi | [S101] |
| EU092849 | | San | [S101] |
| EU092850-EU092853 | | Dama | [S101] |
| EU092854-EU092860 | | San | [S101] |
| EU092861-EU092869 | | SWB | [S101] |
| EU092870-EU092877 | | SEB | [S101] |
| EU092878-EU092888 | | Laal | [S101] |
| EU092889-EU092907 | | Sara | [S101] |
| EU092908-EU092931 | | No information | [S101] |
| EU092932 | | Makrani | [S101] |
| EU092933 | | Makrani | [S101] |
| EU092934 | | Brahui | [S101] |
| EU092935 | | Makrani | [S101] |
| EU092936-EU092966 | | No information | [S101] |
| EU273476-EU273483 | | Pygmy Baka | [S102] |
| EU273484 | | Pygmy Bakola | [S102] |
| EU273485 | | Pygmy Bakola | [S102] |
| EU273486 | | Bantu Benga | [S102] |
| EU273487 | | Bantu Fang | [S102] |
| EU273488 | | Bantu Fang | [S102] |
| EU273489 | | Bantu Galoa | [S102] |
| EU273490 | | Bantu Eshira | [S102] |
| EU273491 | | Bantu Eshira | [S102] |
| EU273492 | | Bantu Akele | [S102] |
| EU273493 | | Bantu Makina | [S102] |
| EU273494 | | Bantu Obamba | [S102] |
| EU273495 | | Bantu Punu | [S102] |
| EU273496 | | Bantu Punu | [S102] |
| EU273497 | | Bantu Shake | [S102] |
| EU273498 | | Bantu Ateke | [S102] |
| EU273499 | | Bantu Mitsogo | [S102] |
| EU273500 | | Bantu | [S102] |
| EU273501 | | Baka Pygmy | [S102] |
| EU273502 | | Duma | [S102] |
| EF556148-EF556165 | | No information | [S103] |
| EF556166 | | Ethiopian Jew | [S103] |
| EF556167-EF556170 | | No information | [S103] |
| EF556171 | | Yemenite Jew | [S103] |
| EF556172 | | No information | [S103] |
| EF556173 | | Ethiopian Jew | [S103] |
| EF556174 | | Ethiopian Jew | [S103] |
| EF556175-EF556196 | | No information | [S103] |
| AY495090-AY495330 | | Caucasian | [S104] |
| DQ304897-DQ305036 | | African American | [S105] |
| DQ282387-DQ282511 | | U.S. Hispanic | [S105] |
| ***Bos taurus*** | | | |
| NC_006853 | N/A | | [S5] |
| AF492351 | N/A | | [S106] |
| EU177867 | isolate Bos18 | | [S107] |
| EU177866 | isolate Bos3 | | [S107] |
| EU177865 | isolate Bos39 | | [S107] |
| EU177864 | isolate Bos38 | | [S107] |
| EU177863 | isolate Bos36 | | [S107] |
| EU177862 | isolate Bos35 | | [S107] |
| EU177861 | isolate Bos22 | | [S107] |
| EU177860 | isolate Bos15 | | [S107] |
| EU177859 | isolate Bos13 | | [S107] |
| EU177858 | isolate Bos12 | | [S107] |
| EU177857 | isolate Bos16 | | [S107] |
| EU177856 | isolate Bos41 | | [S107] |
| EU177855 | isolate Bos23 | | [S107] |
| EU177854 | isolate Bos8 | | [S107] |
| EU177853 | isolate Bos10 | | [S107] |
| EU177852 | isolate GR68 | | [S107] |
| EU177851 | isolate Bos21 | | [S107] |
| EU177850 | isolate Bos5 | | [S107] |
| EU177849 | isolate GR316 | | [S107] |
| EU177848 | isolate Bos37 | | [S107] |
| EU177847 | isolate Bos24 | | [S107] |
| EU177846 | isolate Bos17 | | [S107] |
| EU177845 | isolate Bos7 | | [S107] |
| EU177844 | isolate Bos9 | | [S107] |
| EU177843 | isolate Bos4 | | [S107] |
| EU177842 | isolate Bos2 | | [S107] |
| EU177841 | isolate Bos6 | | [S107] |
| EU177840 | isolate Bos19 | | [S107] |
| EU177839 | isolate Bos11 | | [S107] |
| EU177838 | isolate Bos14 | | [S107] |
| EU177837 | isolate Bos43 | | [S107] |
| EU177836 | isolate Bos42 | | [S107] |
| EU177835 | isolate Bos40 | | [S107] |
| EU177834 | isolate Bos54 | | [S107] |
| EU177833 | isolate Bos53 | | [S107] |
| EU177832 | isolate Bos52 | | [S107] |
| EU177831 | isolate Bos27 | | [S107] |
| EU177830 | isolate Bos20 | | [S107] |
| EU177829 | isolate Bos28 | | [S107] |
| EU177828 | isolate Bos48 | | [S107] |
| EU177827 | isolate Bos31 | | [S107] |
| EU177826 | isolate Bos30 | | [S107] |
| EU177825 | isolate Bos26 | | [S107] |
| EU177824 | isolate Bos25 | | [S107] |
| EU177823 | isolate Bos1 | | [S107] |
| EU177822 | isolate Bos29 | | [S107] |
| EU177821 | isolate Bos45 | | [S107] |
| EU177820 | isolate Bos47 | | [S107] |
| EU177819 | isolate Bos46 | | [S107] |
| EU177818 | isolate Bos34 | | [S107] |
| EU177817 | isolate Bos33 | | [S107] |
| EU177816 | isolate Bos44 | | [S107] |
| EU177815 | isolate Bos32 | | [S107] |
| DQ124418 | isolate H16 | | DS |
| DQ124417 | isolate H15 | | DS |
| DQ124416 | isolate H14 | | DS |
| DQ124415 | isolate H13 | | DS |
| DQ124414 | isolate H12 | | DS |
| DQ124413 | isolate H11 | | DS |
| DQ124412 | isolate H10 | | DS |
| DQ124411 | isolate H9 | | DS |
| DQ124410 | isolate H8 | | DS |
| DQ124409 | isolate H7 | | DS |
| DQ124408 | isolate H6 | | DS |
| DQ124407 | isolate H5 | | DS |
| DQ124406 | isolate H4 | | DS |
| DQ124405 | isolate H3 | | DS |
| DQ124404 | isolate H2 | | DS |
| DQ124402 | isolate FC16 | | DS |
| DQ124401 | isolate FC15 | | DS |
| DQ124400 | isolate FC14 | | DS |
| DQ124399 | isolate FC13 | | DS |
| DQ124398 | isolate FC12 | | DS |
| DQ124397 | isolate FC11 | | DS |
| DQ124396 | isolate FC10 | | DS |
| DQ124395 | isolate FC9 | | DS |
| DQ124394 | isolate FC8 | | DS |
| DQ124393 | isolate FC7 | | DS |
| DQ124392 | isolate FC6 | | DS |
| DQ124391 | isolate FC5 | | DS |
| DQ124390 | isolate FC4 | | DS |
| DQ124389 | isolate FC3 | | DS |
| DQ124388 | isolate FC2 | | DS |
| DQ124387 | isolate FC1 | | DS |
| DQ124386 | isolate KC16 | | DS |
| DQ124385 | isolate KC15 | | DS |
| DQ124384 | isolate KC14 | | DS |
| DQ124383 | isolate KC13 | | DS |
| DQ124382 | isolate KC12 | | DS |
| DQ124381 | isolate KC11 | | DS |
| DQ124380 | isolate KC10 | | DS |
| DQ124379 | isolate KC9 | | DS |
| DQ124378 | isolate KC8 | | DS |
| DQ124377 | isolate KC7 | | DS |
| DQ124376 | isolate KC6 | | DS |
| DQ124375 | isolate KC5 | | DS |
| DQ124374 | isolate KC4 | | DS |
| DQ124373 | isolate KC3 | | DS |
| DQ124372 | isolate KC2 | | DS |
| DQ124371 | isolate KC1 | | DS |
| AY676873 | isolate 32027 | | DS |
| AY676872 | isolate 32026 | | DS |
| AY676871 | isolate 32025 | | DS |
| AY676870 | isolate 32024 | | DS |
| AY676869 | isolate 32023 | | DS |
| AY676868 | isolate 32022 | | DS |
| AY676867 | isolate 32021 | | DS |
| AY676866 | isolate 32020 | | DS |
| AY676865 | isolate 32019 | | DS |
| AY676864 | isolate 32018 | | DS |
| AY676863 | isolate 32017 | | DS |
| AY676862 | isolate 32016 | | DS |
| AY676861 | isolate 26368 | | DS |
| AY676860 | isolate 25651 | | DS |
| AY676859 | isolate 23854 | | DS |
| AY676858 | isolate 22130 | | DS |
| AY676857 | isolate 18905 | | DS |
| AY676856 | isolate 17605 | | DS |
| AY676855 | isolate 16683 | | DS |
| AY526085 | N/A | | DS |
| AB074968 | haplotype:JBC8 | | DS |
| AB074967 | haplotype:JBC7 | | DS |
| AB074966 | haplotype:JBC6 | | DS |
| AB074965 | haplotype:JBC5 | | DS |
| AB074964 | haplotype:JBC3 | | DS |
| AB074963 | haplotype:JBC2 | | DS |
| AB074962 | haplotype:JBC1 | | DS |
| V00654 | N/A | | [S108] |
| ***Canis familiaris*** | | | |
| NC_002008 | Sapsaree | | [S19] |
| DQ480502 | Jamthund | | [S20] |
| DQ480501 | Swedish Elkhound | | [S20] |
| DQ480500 | Shetland Sheepdog | | [S20] |
| DQ480499 | Siberian Husky | | [S20] |
| DQ480498 | Miniature Schnauzer | | [S20] |
| DQ480497 | West Highland White Terrier | | [S20] |
| DQ480496 | Irish Soft Coated Wheaten Terrier | | [S20] |
| DQ480495 | Cocker Spaniel | | [S20] |
| DQ480494 | Poodle | | [S20] |
| DQ480493 | Black Russian Terrier | | [S20] |
| DQ480492 | Jamthund | | [S20] |
| DQ480491 | Irish Setter | | [S20] |
| DQ480490 | Flat Coated Retriever | | [S20] |
| DQ480489 | German Shepherd | | [S20] |
| AY656755 | Sapsaree | | DS |
| AY656754 | Chinese Crested | | DS |
| AY656753 | Irish Setter | | DS |
| AY656752 | Standard Schnauzer | | DS |
| AY656751 | Gordon Setter | | DS |
| AY656750 | Leonberger | | DS |
| AY656749 | Saint Bernard | | DS |
| AY656748 | Airedale Terrier | | DS |
| AY656747 | Welsh Springer Spaniel | | DS |
| AY656746 | Standard Schnauzer | | DS |
| AY656745 | English Springer Spaniel | | DS |
| AY656744 | English Springer Spaniel | | DS |
| AY656743 | Saint Bernard | | DS |
| AY656742 | Old English Sheepdog | | DS |
| AY656741 | Irish Setter | | DS |
| AY656740 | Kerry Blue Terrier | | DS |
| AY656739 | Poodle | | DS |
| AY656738 | Jack Russell Terrier | | DS |
| AY656737 | Basenji | | DS |
| AY729880 | Unknown | | DS |
