# Supplementary material for: Reconstructing Mammalian Phylogenies: A Detailed Comparison of the Cytochrome b and Cytochrome Oxidase Subunit I Mitochondrial Genes
Source: PLoS One. 2010 Nov 30;5(11):e14156. doi: 10.1371/journal.pone.0014156 (PMC2994770; doi:10.1371/journal.pone.0014156)
Supplement: Table S3 — The statistical results from the Maximum Likelihood phylogenetic trees calculated using MrBayes [S111–112] from 15,002 trees sampled. Tree length, six reversible substitution rates, four stationary state frequencies (pi), the shape of the gamma distribution (α) and the proportion of invariable sites (pinvar) are displayed. (0.07 MB DOC) [file pone.0014156.s018.doc]

|  |  |  | **95 % Credibility Interval** | |  |
| --- | --- | --- | --- | --- | --- |
| **Parameter** | **Mean** | **Variance** | **Lower** | **Upper** | **Median** |
| **COI** |  |  |  |  |  |
| **TL{all}** | 59.71051 | 2.341455 | 56.876 | 62.723 | 59.749 |
| **r(A<->C){all}** | 0.009009 | 0.000001 | 0.0078 | 0.010724 | 0.008918 |
| **r(A<->G){all}** | 0.557012 | 0.000118 | 0.537907 | 0.583205 | 0.557172 |
| **r(A<->T){all}** | 0.021699 | 0.000001 | 0.0196 | 0.023421 | 0.021668 |
| **r(C<->G){all}** | 0.028674 | 0.000009 | 0.023133 | 0.034436 | 0.028673 |
| **r(C<->T){all}** | 0.341011 | 0.000108 | 0.317496 | 0.35843 | 0.341721 |
| **r(G<->T){all}** | 0.042596 | 0.000017 | 0.035519 | 0.050746 | 0.042566 |
| **pi(A){all}** | 0.354282 | 0.000051 | 0.339114 | 0.366266 | 0.355548 |
| **pi(C){all}** | 0.284318 | 0.000021 | 0.275986 | 0.292859 | 0.283857 |
| **pi(G){all}** | 0.060271 | 0.000002 | 0.057173 | 0.063153 | 0.060364 |
| **pi(T){all}** | 0.301129 | 0.000018 | 0.294624 | 0.310854 | 0.300565 |
| **alpha{all}** | 0.409732 | 0.000032 | 0.399072 | 0.420895 | 0.409482 |
| **pinvar{all}** | 0.453507 | 0.000131 | 0.431053 | 0.47517 | 0.453348 |
|  |  |  |  |  |  |
| **Cyt *b*** |  |  |  |  |  |
| **TL{all}** | 59.66446 | 2.882398 | 56.701 | 63.177 | 59.532 |
| **r(A<->C){all}** | 0.010753 | 0.000001 | 0.009232 | 0.012196 | 0.010722 |
| **r(A<->G){all}** | 0.454693 | 0.000154 | 0.428025 | 0.475739 | 0.454002 |
| **r(A<->T){all}** | 0.033873 | 0.000002 | 0.031122 | 0.03759 | 0.033745 |
| **r(C<->G){all}** | 0.02804 | 0.000008 | 0.022769 | 0.03392 | 0.028209 |
| **r(C<->T){all}** | 0.38207 | 0.000128 | 0.360897 | 0.407648 | 0.381063 |
| **r(G<->T){all}** | 0.090571 | 0.000042 | 0.078293 | 0.102555 | 0.090911 |
| **pi(A){all}** | 0.386998 | 0.000068 | 0.368706 | 0.404746 | 0.386619 |
| **pi(C){all}** | 0.373289 | 0.000042 | 0.360024 | 0.388501 | 0.373981 |
| **pi(G){all}** | 0.044744 | 0.000001 | 0.042539 | 0.046934 | 0.044564 |
| **pi(T){all}** | 0.194969 | 0.000011 | 0.188204 | 0.200709 | 0.195777 |
| **alpha{all}** | 0.432066 | 0.000048 | 0.418708 | 0.44579 | 0.432073 |
| **pinvar{all}** | 0.294149 | 0.000228 | 0.264054 | 0.323522 | 0.294033 |
