# Supplementary material for: Reconstructing Mammalian Phylogenies: A Detailed Comparison of the Cytochrome b and Cytochrome Oxidase Subunit I Mitochondrial Genes
Source: PLoS One. 2010 Nov 30;5(11):e14156. doi: 10.1371/journal.pone.0014156 (PMC2994770; doi:10.1371/journal.pone.0014156)
Supplement: Table S5 — The statistical results from the ROC curve (Figure S3). Calculated in SPSS 17.0.0. (0.03 MB DOC) [file pone.0014156.s020.doc]

| **Test Result Variable(s)** |  | | | **Asymptotic 99% Confidence Interval** | |
| --- | --- | --- | --- | --- | --- |
|  | **Area** | **Std. Error^a^** | **Asymptotic Sig.^b^** | **Lower Bound** | **Upper Bound** |
| **Cyt *b*** | 1.000 | .000 | .000 | 1.000 | 1.000 |
| **COI** | 1.000 | .000 | .000 | 1.000 | 1.000 |
| The test result variable(s): Cyt *b*, COI has at least one tie between the positive actual state group and the negative actual state group. Statistics may be biased. | | | | | |
| a. Under the nonparametric assumption | | | | | |
| b. Null hypothesis: true area = 0.5 | | | | | |
